# Surface Morphology and Degradation of Poly[(*R*)-3-Hydroxybutyrate]-*block*-Poly(ε-Caprolactone) and Poly[(*R*)-3-Hydroxybutyrate]-*block*-Poly(l-Lactide) Biodegradable Diblock Copolymers

**DOI:** 10.3390/polym17111558

**Published:** 2025-06-03

**Authors:** Ayan Bartels-Ellis, Senri Hayashi, Tomohiro Hiraishi, Takeharu Tsuge, Hideki Abe

**Affiliations:** 1 Bioplastic Research Team, RIKEN Center for Sustainable Resource Science, 21 Hirosawa, Wako, Saitama 351-0198, Japan; ayan.bartels-ellis@riken.jp (A.B.-E.); senri.hayashi@riken.jp (S.H.); thiraish@riken.jp (T.H.); 2 Department of Materials Science and Engineering, Institute of Science Tokyo, 4259 Nagatsuta, Midori-ku, Yokohama 226-8502, Japan; tsuge.t.aa@m.titech.ac.jp

**Keywords:** biodegradable polymer, block copolymer, crystallization, surface morphology, PHA, poly-3-hydroxybutyrate, polylactic acid, polycaprolactone, enzymatic degradation, AFM

## Abstract

Bacterially produced poly[(*R*)-3-hydroxybutyrate] (P3HB) was subjected to an alcoholysis reaction to produce low-molecular-weight (*M*_n_ ≈ 10,000 g mol^−1^) hydroxy-terminated P3HB (LMPHB). Using diethyl zinc as a catalyst, LMPHB was reacted with the cyclic monomers ε-caprolactone and l-lactide in separate ring-opening polymerization (ROP) reactions to produce PHB-*b*-PCL (PHBCL) and PHB-*b*-PLA (PHBLA) AB-type crystalline–crystalline diblock copolymers with varying PCL and PLA block lengths. ^1^H NMR and GPC were used to confirm the structure of the polymers. DSC was used to measure the thermal properties as well as assessing crystallization. A single-shifting *T*_g_ for PHBLA showed the two blocks to be miscible in the melt. The TGA results indicate enhanced thermal stability over the homopolymer P3HB. A study of the crystallization was undertaken by combining WAXD, a second DSC heating regime, and POM. POM showed that the crystallization in PHBCL to be dependent on the crystallization temperature more so than PHBLA, whose composition appeared to be the more definitive factor determining the spherulitic morphology. The results informed the crystallization temperatures used in the production of the melt-crystallized thin films that were imaged using AFM. AFM images showed unique surface morphologies dependent on the diblock copolymer composition, block length, and crystallization temperature. Finally, the enzymatic degradation studies showed these unique surface morphologies to influence how these block copolymers were degraded by enzymes.

## 1. Introduction

Poly[(*R*)-3-hydroxybutyrate) (P3HB), poly(ε-caprolactone) (PCL), and poly(l-lactide) (PLA) represent some of the most widely researched biodegradable polymers. Whilst their application to date has mostly been limited to the biomedical sphere, their impressive mechanical properties and ability to degrade in the natural environment means that they can be proposed as environmentally conscious alternatives to replace the current petroleum-derived synthetic plastics that cause environmental pollution [1,2,3,4]. Despite this, the properties of these materials have not been explored, notably the brittleness of both P3HB and PLA, which prevents their broader application. Numerous approaches are being considered to tackle this issue, including polymer blending, additives, and chemical modification [5,6,7,8]. These approaches offer potential improvements to both the mechanical properties as well as the degradation character of biodegradable polymers.

Block copolymerization is a type of chemical modification that covalently joins two or more polymers [9]. In 1993, Reeve et al. published their paper on the synthesis of PHBCL and PHBLA diblock copolymers using a triethyl aluminum (AlEt_3_) catalyst to create a low-molecular-weight P3HB (LMPHB) macroinitiator, PHB-O-AlEt_2_, that was used in the ROP of ε-caprolactone (ε-CL) and l-lactide cyclic monomers [10]. It was found that, for PHBLA-type diblock copolymers, P3HB and PLA chain segments were miscible when in the melt state; additionally, both crystallinity and phase separation values were lower than those of the respective P3HB and PLA blend. More authors have since paid attention to the synthesis of block copolymers based on these materials; however, most have focused on their use as compatibilizers in blends [11,12,13,14]. Given the well-documented relationship between the crystallinity and surface morphology of biodegradable polymers with enzyme interaction, and as a result biodegradation, there is a clear need for studies investigating how crystallinity and surface morphology manifest in biodegradable block copolymers.

The AB-type diblock copolymer is the simplest type of block copolymer, concerning two block segments A and B, covalently joined at a single point. When two crystallizable blocks are used for segments A and B, the crystallization in the copolymer becomes complex, with the difference in the melting temperature (*T*_m_) of the A and B blocks being a crucial factor [15]. P3HB (*T*_m_ = 170–180 °C), combined with either PCL (*T*_m_ = 60–65 °C, separated *T*_m_) or PLA (*T*_m_ = 170–200 °C, close *T*_m_), provides a useful case to investigate crystallinity in crystalline–crystalline diblock copolymers. Hamley et al. investigated crystallization in PLLA-*b*-PCL diblock copolymers, finding that sequential crystallization could be used to influence the overall crystal structure, with PLLA forming spherulites at higher temperatures and PCL crystallizing within the PLLA spherulite and rearranging the lamellar structure after cooling to below the *T*_m_ of PCL [16]. As has been highlighted by others, the enzymatic degradation of biodegradable polymers is heavily impacted by spherulitic and lamellar morphologies [17,18,19,20,21]. The impact of complex crystallization, possible by biodegradable crystalline–crystalline diblock copolymers, on biodegradation may thus provide a potential route to regulate the biodegradation of biodegradable polymers.

The difference between biodegradable and non-biodegradable polymers is the ability to be degraded by microorganisms, more specifically the enzymes secreted by these microorganisms. Since microorganisms have not adapted to degrade non-biodegradable polymers, these materials accumulate as microplastics in the environment. The biodegradation of biodegradable polymers in the environment is a multi-step process; however, the rate-limiting step is largely thought to be the enzymatic degradation of microplastics by enzymes [22]. P3HB is degraded by PHB depolymerase enzymes, whereas PCL is degraded primarily by lipases. PLA is known to be degraded by proteinase K, with the work by Iwata and Doi as well as that by Tsuji and Miyauchi showing amorphous regions to be preferentially degraded over crystalline regions, with the free amorphous region outside of the spherulite to be degraded faster than the interlamellar amorphous region found inside the spherulite [18,23]. Similar work into the enzymatic degradation of P3HB crystals by PHB depolymerase has shown the disordered chain-packing region to be preferentially degraded [24]. Thus, the literature has shown that the degradation rate of both P3HB and PLA by their respective enzymes can be manipulated by changes in their crystallinity.

This study synthesized and investigated PHB-*b*-PCL (PHBCL) and PHB-*b*-PLA (PHBLA) diblock copolymers of varying PCL or PLA block lengths (roughly in the ratios 2:1, 1:1, and 1:2 of PHB:PCL/PLA). The thermal properties were assessed using differential scanning calorimetry (DSC) and thermal gravimetric analysis (TGA). Crystallization at different isothermal crystallization temperatures (*T*_c_) and any spherulitic morphologies were observed using polarized optical microscopy (POM). Surface morphology was investigated using melt-crystallized thin films that were observed using atomic force microscopy (AFM). Using PHB depolymerase, lipase, and proteinase K, the effects of enzymatic degradation on the surface morphology were also observed by AFM.

By understanding the influence that polymer crystallinity has on both the physical properties and degradation of biodegradable polymers, this study will help to elucidate the crystalline structure, surface morphology, and enzymatic degradation of P3HB-based biodegradable block copolymers and how these factors are influenced by both the block composition and length.

## 2. Materials and Methods

### 2.1. Materials

Bacterially produced P3HB was kindly provided by the now-defunct Imperial Chemical Industries PLC (London, UK). P3HB was purified by dissolution in chloroform, reprecipitation in n-hexane, followed by vacuum filtration and drying in vacuo overnight at 40 °C. l-lactide (Purac Co., Amsterdam, The Netherlands) was recrystallized from a toluene solution at 65 °C. ε-CL was dried over calcium hydride and distilled under pressure before use.

### 2.2. Synthesis of PHBCL and PHBLA Diblock Copolymers

**Scheme 1:**
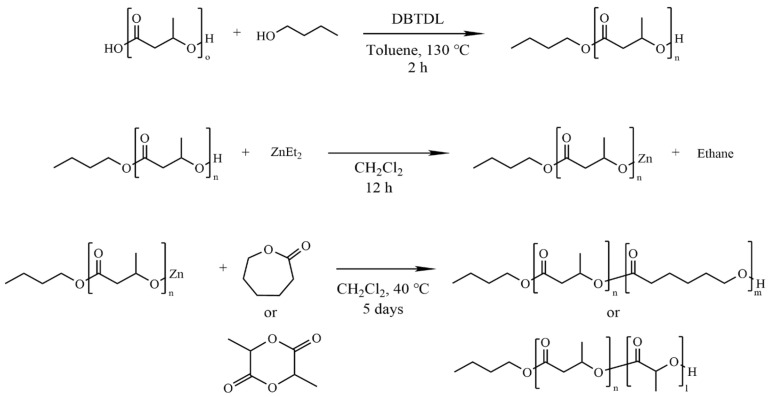
Three-step synthesis route for PHBCL and PHBLA diblock copolymers.

Scheme 1 shows the synthesis route used to attain the PHBCL and PHBLA diblock copolymers of varying PCL/PLA block lengths. In brief, an alcoholysis reaction was used to produce stereoisomerically pure LMPHB oligomers from high-molecular-weight P3HB produced originally through bacterial fermentation. LMPHB was subsequently activated via reaction with diethyl zinc (ZnEt_2_), which acted as a catalyst and produced the reactive intermediate (LMPHB-O-ZnEt_2_) that was used to initiate the ROP of either ε-CL or l-lactide cyclic monomers.

#### 2.2.1. Synthesis of LMPHB Initiator

Purified P3HB (7.5 g) was placed into a 1 L round-bottom flask containing a magnetic stirrer, along with n-butanol (75 mL), toluene (750 mL), and 0.2 M dibutyltin dilaurate (DBTDL)/toluene (1200 µL). This flask was then placed into a preheated oil bath set to 130 °C, with a distillation column fitted to allow for reflux conditions. The reaction was stopped after 2 h. The reaction solution was precipitated in a 10-times volume excess of hexane, filtered via vacuum filtration onto filter paper, and subsequently dried in vacuo overnight at 40 °C. LMPHB was analyzed using ^1^H NMR and GPC to obtain the chemical structure and molecular weight before being used in the synthesis of PHB-*b*-PCL and PHB-*b*-PLA diblock copolymers.

#### 2.2.2. ROP of ε-CL and l-Lactide Cyclic Monomers

To prepare the activated LMPHB initiator, a 300 mL 2-neck round bottom flask with a magnetic stirrer was rigorously dried and purged with nitrogen under a reduced atmosphere; great care to prevent contact with the atmosphere was taken at all stages of this reaction. LMPHB (7 g) was added to the flask along with CHCl_2_ (140 mL). After the polymer had completely dissolved, the flask was placed in an ice bath for the addition of the ZnEt_2_ catalyst (0.055 M); then, the flask was sealed and removed from the ice bath, and the reaction was left to proceed overnight at room temperature. Following this, the reaction vessel was placed under reduced pressure to allow for the evaporation of the solvent. After the complete evaporation of the solvent, the flask was sealed and stored in a glovebox.

Into a 100 mL round-bottom flask with a magnetic stirrer, the activated LMPHB initiator (1 g) and CHCl_2_ (20 mL) were added, allowing all the LMPHB initiator to dissolve before the addition of ε-CL or l-lactide monomer. Depending on the desired PCL or PLA block length, the amount of ε-CL or l-lactide added varied, such that, for the diblock copolymer with PHB:PCL/PLA ratio equal to 2:1, ε-CL (0.22 M)/l-lactide (0.17 M) was added; for 1:1, ε-CL (0.44 M)/l-lactide (0.35 M); and for 1:2, ε-CL (0.88 M)/l-lactide (0.69 M). After the addition of the monomer, the reaction vessel was tightly sealed and removed from the glovebox to a preheated oil bath set to 40 °C and left for 5 days to allow for the ROP reaction to proceed. The reaction was terminated by adding a small volume of MeOH to the reaction vessel after breaking the seal. CHCl_3_ was added to dissolve any solidified polymer material before the precipitation of the polymer solution in a 10-times volume excess of MeOH. The precipitate was obtained through vacuum filtration and, then, dried under vacuum at 40 °C. To remove residual Zn compounds, the polymer solutions in CHCl_3_ were washed 3 times using an equal volume of 0.1 M acetic acid solution and MeOH precipitated in excess.

### 2.3. Structural Analysis (^1^H NMR and GPC)

The ^1^H NMR spectra for the synthesized polymers were recorded with a Varian NMR System 500 MHz spectrometer (Agilent Technologies, Inc., Santa Clara, CA, United States) at 25 °C, using polymers as 1% (*w*/*v*) CDCl_3_ solutions containing tetramethylsilane (TMS) as the internal standard. Chemical shifts in parts per million (ppm) were referenced relative to TMS, with the NMR being conducted in a 0.0–12.5 ppm chemical shift window. NMR data were analyzed using the MestReNova software.

The molecular weights for the synthesized polymers were measured using a Shimadzu Nexera GPC (Shimadzu Co., Kyoto, Japan) and RID-20A refractive index detector with Shodex KF-806 M and KF-802 columns (Resonac Co., Tokyo, Japan) at 40 °C. The samples were prepared by dissolving polymers in HPLC-grade CHCl_3_ at a 1% (*w*/*v*) concentration. HPLC-grade CHCl_3_ was used as the eluent at a flow rate of 0.8 mL/min, and 9 polystyrene standards containing 1.32 × 10^3^, 3.25 × 10^3^, 1.01 × 10^4^, 2.85 × 10^4^, 6.60 × 10^4^, 1.56 × 10^5^, 4.60 × 10^5^, 1.07 × 10^6^, and 3.15 × 10^6^ number-average molecular weights were used to generate the calibration curve.

### 2.4. Thermal Analysis (DSC, TGA and WAXD)

The thermal properties were measured using a DSC8500 calorimeter (PerkinElmer Inc., Yokohama, Japan) under helium flow, with indium being used as a calibration standard for the temperature and heat capacity measurements. Aluminum pans were used to encapsulate 3–5 mg of the polymer sample, with an empty pan being used as the reference. The heating regime consisted of an initial heating (1st heating) in the range of −100–200 °C at a heating rate of 20 °C/min; after holding at 200 °C for 1 min, the samples were rapidly quenched to −100 °C before being exposed to a second heating (2nd heating) in the range of −100–200 °C at 20 °C/min. The glass transition temperature (*T*_g_) was taken as the midpoint of the heat capacity change seen in the 2nd heating.

A separate heating regime was used to analyze crystallization from the melt due to the formation of crystallization peaks that were obstructed when the sample was rapidly quenched from the melt. In this second heating regime, the sample was heated in the range of −100–200 °C at a heating rate of 20 °C/min (1st heating); after holding at 200 °C for 1 min, the samples were cooled to −100 °C at a cooling rate of −10 °C/min (cooling), and the samples were then subsequently heated in the range of −100–200 °C at a heating rate of 10 °C/min (2nd heating). The cooling stage was used to extract data on the crystallization temperature (*T*_c_) and the enthalpy of fusion (Δ*H*_c_).

The thermal degradation behavior was measured using a TG/DTA7200 (Hitachi High-Tech Science Co., Tokyo, Japan) under nitrogen flow. A total of 3–5 g of the sample was weighed in an aluminum pan before being loaded onto the balance and heated from room temperature to 500 °C at a heating rate of 10 °C/min, using an empty pan as the reference.

### 2.5. WAXD Measurements

Wide-angle X-ray diffraction (WAXD) patterns of copolymer samples were recorded at 25 °C on a Rigaku RINT 2500 system (Rigaku Holdings Co., Tokyo, Japan) using a nickel-filtered Cu Kα radiation (λ = 0.154 nm; 40 kV; 100 mA) in the 2θ range from 4 to 60° at a scanning speed of 2.0 °/min. The crystallinity of the copolymer samples was determined using diffraction intensity data, in accordance with Vonk’s method [25].

### 2.6. POM Observation

POM observation was performed using a BX53 Upright Microscope (Olympus Co., Tokyo, Japan) equipped with a THMS600 temperature-controlled microscope stage (Linkam Scientific Instruments, Salford, UK). A small amount of the sample (2–3 mg) was sandwiched between two glass cover slides and heated to 200 °C at a 20 °C/min heating rate on the heating stage. The samples were held at 200 °C for 30 s whilst gently pressing the upper cover glass to produce a thin sample for observation and to prevent air bubble formation; following this, the sample was rapidly cooled to the desired *T*_c_ and imaged.

### 2.7. Melt-Crystallized Thin-Film Sample Preparation

Using a MSD-200 desktop type spin coater (JAPAN CREATE Co., Ltd, Saitama, Japan), 20 µL of 2% (*w*/*v*) CHCl_3_ polymer solution was deposited onto a glass base substrate of 18 mm × 18 mm dimensions that was already spinning at 5000 rpm. Following deposition, rotation was maintained for 30–60 s to facilitate the formation of a uniform thin film of polymer as well as the evaporation of residual solvent. The thin-film samples produced through spin coating were subsequently melted using a pre-heated hot plate set to 200 °C for up to 3 min. Following this, the samples were quickly transferred to a separate pre-heated hot plate set to the desired *T*_c_ and left for 3–4 days before being removed from the hot plate and left to cool to room temperature for a minimum of 24 h before use in the AFM observation.

### 2.8. AFM Observation

The AFM observation of the melt-crystallized thin-film surface morphology was undertaken with a AFM5200S (Hitachi High-Tech Co., Tokyo, Japan), using the dynamic force mode (tapping mode) at room temperature using a silicon cantilever back-coated with aluminum, SI-DF-3 (Hitachi High-Tech Co., Tokyo, Japan) with a length of 450 μm, spring constant of 1.5 N/m, and peak frequency of 26 kHz. Height and phase images were obtained simultaneously using a scan rate of 1.0 Hz.

### 2.9. Enzymatic Degradation

Table 1 summarizes the enzymes and conditions for enzymatic degradation utilized to target the degradation of the different biodegradable polymers utilized in this study, PHB, PCL, and PLA. All degradations were undertaken at 35 °C, using an upturned Petri dish to mitigate the effects of the evaporation of the enzyme solution from the thin-film surface.

Extracellular PHB depolymerase from *Ralstonia pickettii* T1 (previously named *Alcaligenes faecalis* T1), purified to electrophoretic homogeneity by the method of Tan et al. and stored at 0–4 °C, was used for enzymatic degradation targeting the P3HB of melt-crystallized polymer thin films [26]. A total of 100 µL of 0.1 M potassium phosphate buffer (pH 7.4) containing 1 µg/mL of PHB depolymerase was pipetted onto the surface of melt-crystallized thin films and left for 40 min at room temperature, after which the enzyme solution was removed by washing the surface of the film with distilled water. The enzymatically degraded thin film was then left to dry under ambient conditions for up to 24 h prior to AFM observation. For the targeted degradation of the PCL block segments, 50 µL of 0.1 M potassium phosphate buffer (pH 7.4) containing 1 mg/mL of Lipase PS Amano SD from *Burkholderia cepacia* was pipetted onto the surface of PHB-*b*-PCL samples and left for 30 min. Following degradation, the film was washed with distilled water to remove the enzyme and left to dry in air, as previously stated. The PLA block segments were targeted via pipetting 100 μL of 0.2 mg/mL of proteinase K from *Tritachium album* in 50 mM tris-HCl buffer solution (pH 8.5) onto the surface of PHB-*b*-PLA thin films. Degradation using proteinase K enzymatic solutions was allowed to proceed for 120 min. After this time elapsed, the degraded thin films were washed as previously stated.

## 3. Results and Discussion

### 3.1. Synthesis and Characterization of Diblock Copolymers

**Figure 1:**
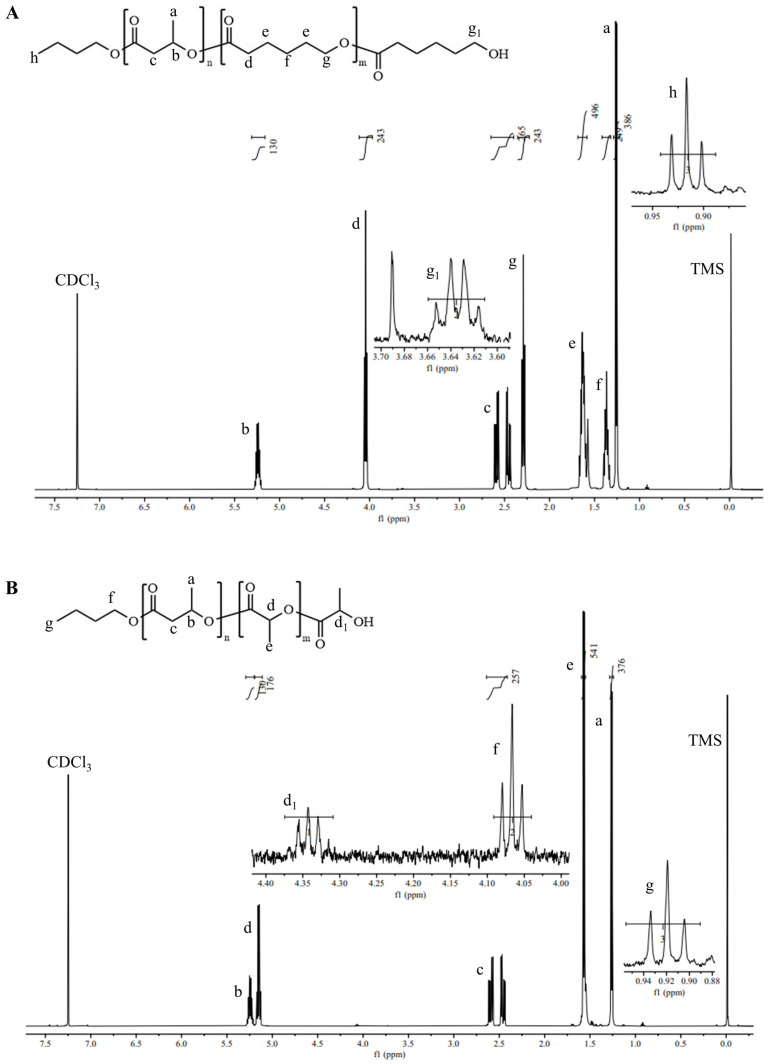
The 500 MHz ^1^H NMR spectrum characterization of (**A**) PHBCL 1.0CL and (**B**) PHBLA 1.0LA in CDCl_3_.

PHB-*b*-PCL (PHBCL) and PHB-*b*-PLA (PHBLA) series of diblock copolymers were synthesized in a three-step reaction. In the first step, high-molecular-weight bacterially produced P3HB (HMPHB) reacted with n-butanol in an alcoholysis reaction using DBTDL to cut the molecular weight. This reaction reduces the molecular weight of HMPHB through a process of random chain scission, with the desired molecular weight being attainable through the manipulation of the reaction time [27]. For the purposes of this study, a number-average molecular weight (*M*_n_) of roughly 10,000 g mol^−1^ was desired, which, through experiments, was found to require a 2 h reaction time. The GPC measurements showed that the original HMPHB had *M*_n_ = 174,000 with a polydispersity (*M*_w_/*M*_n_) of 2.47; after alcoholysis, this was reduced to *M*_n_ = 26,000 with *M*_w_/*M*_n_ = 1.48. The ^1^H NMR chemical shift integration calculations showed LMPHB to have *M*_n_ = 11,000 g mol^−1^; this was calculated by comparing the methylene peak arising from the end-group n-butanol at δ = 4.06 ppm with the peak arising from the main-chain P3HB methine at δ = 5.25 ppm. These peaks can be seen in the ^1^H NMR spectrum of LMPHB, shown in Figure 1. The apparent discrepancy in molecular weight values when measured by GPC and ^1^H NMR is a result of interactions between LMPHB and the GPC column, causing LMPHB to have a larger retention time than would be expected by ^1^H NMR. The reaction yields for the alcoholysis reaction as well as the subsequent ROP reactions are displayed in Table 2.

After LMPHB was successfully produced through alcoholysis with n-butanol using a DBTDL catalyst, the hydroxyl-terminated oligomer was activated by reaction with ZnEt_2_ to produce an intermediate that was then used as the macro-initiator to catalyze the ROP of the cyclic monomers ε-CL or l-lactide. This method of synthesis has been used by others in a ‘one-pot’ living polymerization context; however, the present study was focused on diblock copolymers with varying block lengths required for splitting this into two distinct steps [28]. Table 2 shows the results of the ROP of ε-CL or l-lactide in the production of PHBCL and PHBLA diblock copolymers. The yield of such polymers was in the range of 85–95%. The high yield in combination with the low polydispersity measured by GPC is typical of ROP reactions [29,30,31]. The lower yield of the PHBLA series of diblock copolymers may be a result of the lower conversion of l-lactide compared to ε-CL. Figure 1 shows the ^1^H NMR spectra for PHBCL 1.0CL and PHBLA 1.0LA diblock copolymers. The chemical structures of PHBCL and PHBLA diblock copolymers were confirmed by ^1^H NMR spectroscopy, with the molar ratio of block segments being calculated by chemical shift integration calculations.

**Figure 2:**
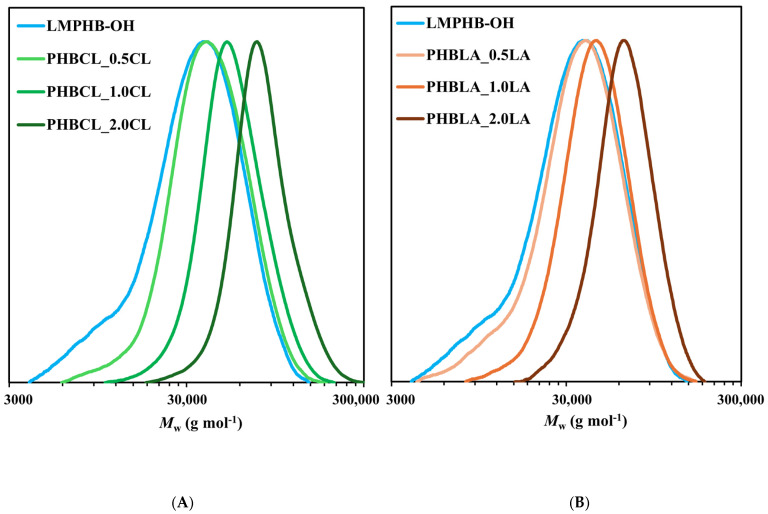
Normalized GPC chromatograms of the (**A**) PHBCL and (**B**) PHBLA diblock copolymers polymers, with the LMPHB initiator included for a reference in each case, using CDCl_3_ as the eluent and a polystyrene standard.

Figure 2 shows the GPC chromatograms for LMPHB and the PHBCL and PHBLA diblock copolymers. The symmetrical unimodal curve that shifts to higher molecular weight values as the length of the PCL/PLA block increases attests to the purity of the produced polymer as well as further confirming the PHB:PCL/PLA block segments’ molar ratio measured by ^1^H NMR. It can also be seen from the chromatograms in combination with data from Table 2 that, as the non-P3HB block segment increases, the polydispersity decreases, such that the highest-molecular-weight polymers, PHBCL 2.0CL and PHBLA 2.0LA, both have a low *M*_w_/*M*_n_ value of 1.16.

### 3.2. Thermal Analysis

#### 3.2.1. DSC (Thermal Properties)

**Figure 3:**
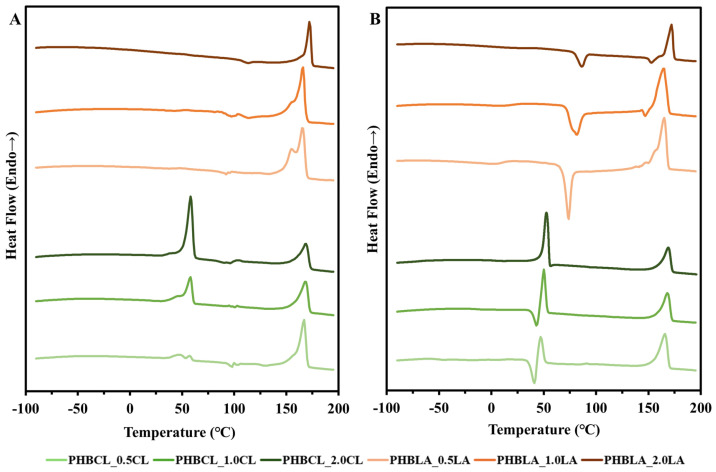
(**A**) First-heating and (**B**) second-heating DSC thermograms showing relative change in heat flux with the temperature for the PHBCL and PHBLA diblock copolymers with varying PCL/PLA block lengths.

The thermal properties of PHBCL and PHBLA were characterized by DSC, with the results compiled in Table 3. To analyze the thermal properties using DSC, the samples were heated in the range of −100–200 °C at a 20 °C/min heating rate, followed by quenching to −100 °C, before heating for a second time in the range of −100–200 °C at 20 °C/min; the corresponding thermograms can be seen in Figure 3. The use of this heating regime was to analyze, primarily, the melting (*T*_m_) and glass transition temperatures (*T*_g_). All diblock copolymers exhibited melting behavior during the first heating scans.

The PHBCL diblock copolymers exhibited two melting peaks corresponding to PCL, *T*_m_ ≈ 60 °C, and P3HB, *T*_m_ ≈ 170 °C, block segments. As the length of the PCL block segment increases, the melting peak for both PCL and P3HB experiences an insignificant shift of 1–2 °C to a higher temperature. On the other hand, the heat of fusion (∆*H*_m_) for the corresponding melting peaks changes such that, with the increase in the PCL block length, P3HB ∆*H*_m_ decreases from 65.8 J g^−1^ in PHBCL 0.5CL to 32.6 J g^−1^ in PHBCL 2.0CL, whilst the opposite trend is seen for PCL ∆*H*_m_, as it increases from 2.0 J g^−1^ in PHBCL 0.5CL to 50.5 J g^−1^ in PHBCL 2.0CL. These results prove the presence of both P3HB and PCL crystalline segments in all PHBCL diblock copolymers, with the increase in the PCL segment length hindering the crystallinity of the P3HB segment. Interestingly, PHBCL 0.5CL showed a double melting peak during the first-heating scan, with this second melting peak appearing as a small shoulder as the PCL segment length increases; there are many possible reasons for such a result, including phenomena such as different lamellar populations producing two distinct melting peaks [32]. The change in the heat capacity used for determining *T*_g_ was hard to identify in the PHBCL diblock copolymers’ second-heating thermograms, meaning that *T*_g_ could not be used to draw conclusions on block miscibility. Despite this, the fact that, regardless of the composition, both PCL and P3HB *T*_m_ values remain relatively constant suggests that the blocks are to some extent immiscible.

The PHBLA diblock copolymers exhibited a single *T*_m_ in the range of 166–172 °C during the first heating. In the case of PHBLA 0.5LA, this was a complex melting peak that could potentially arise from separate PHB and PLA crystal phases. After melting at 200 °C and conducting the second heating, this became a single melting peak. The presence of only one single shifting *T*_g_ for all compositions of PHBLA during the second-heating scan (see Supporting Information, Figure S19), found to be in the range of 10–35 °C, the range of values reported for PHB (0 °C) and PLA (55 °C), provides further evidence to suggest that the PHB and PLA block segments are miscible with each other in the melt. Through the decrease in ∆*H*_m_ as the PLA block length increases, the PHBLA crystallinity can be understood to decrease as the PLA block length increases. This, in combination with the cold crystallization peak that decreases in size as the PLA block length increases, seen in the second-heating runs, suggests that only the PHB block segment is ordering into a crystalline phase during this heating regime.

#### 3.2.2. WAXD

**Figure 4:**
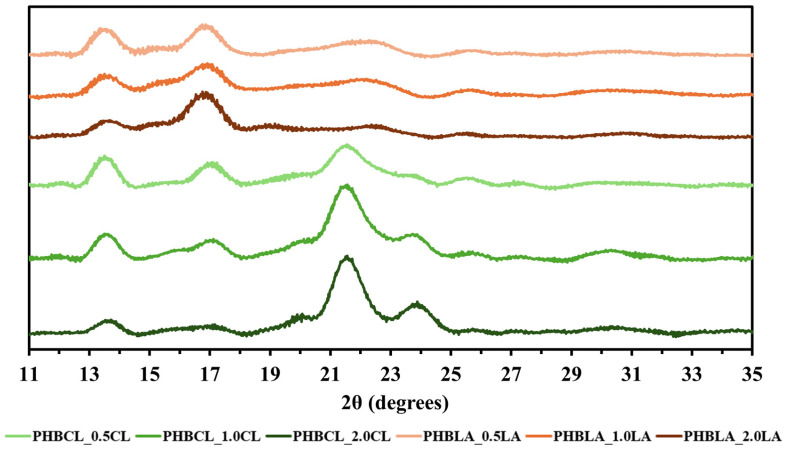
WAXD patterns of the PHBCL and PHBLA diblock copolymers with varying PCL/PLA block lengths; measurements were taken for 2θ values in the range of 4–60° at a scanning speed of 2.0°/min.

Whilst WAXD cannot provide absolute calculations for crystallinity, by comparing peak areas, relative changes in crystallinity amongst the ‘as synthesized’ diblock copolymers of varying block lengths can be understood; the WAXD patterns are shown in Figure 4. Both DSC and WAXD data show the PHBCL series of diblock copolymers to have a higher degree of crystallinity compared to the PHBLA counterparts. This reduction in crystallinity in PHBLA may be a result of the PLA block possessing a higher *T*_g_ value than PCL, potentially limiting the mobility of the PHB blocks, in turn reducing their ability to crystallize. In PHBCL, PHB crystalline regions show two strong peaks at 2θ = 13.7–14.0° and 2θ = 17.2–17.6°, whilst PCL has two strong peaks at 2θ = 21.6–22° and 2θ = 24°, with the latter peak being barely visible in PHBCL 0.5CL. In PHBLA, the peaks for PHB are present, with weak peaks at positions associated with PLA, suggesting that, in PHBLA, crystallinity can be mostly attributed to the PHB block segment.

#### 3.2.3. TGA

**Figure 5:**
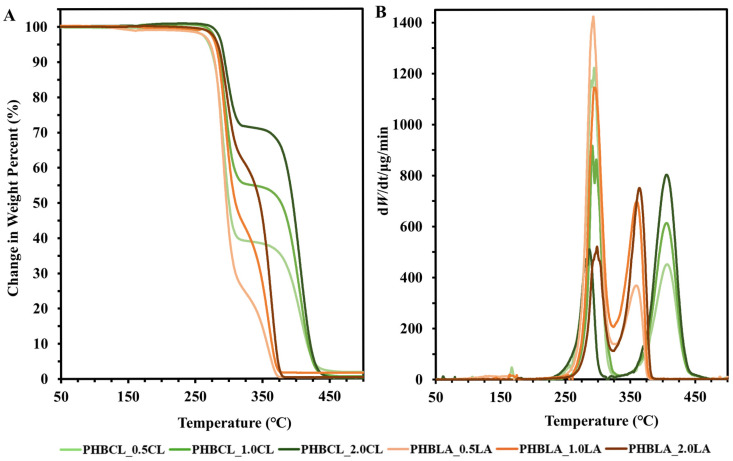
(**A**) TGA and (**B**) DTG curves for the PHBCL and PHBLA diblock copolymers with varying PLA/PCL block lengths in the range of 40–500 °C using a 10 °C/min heating regime.

The thermal degradation properties were analyzed by TGA. Figure 5 shows the TGA and DTG curves during heating in the range of 50–500 °C at a 10 °C/min heating rate under a nitrogen atmosphere. The temperatures of 5 wt.% (*T*_d5%_), 50 wt.% (*T*_d50%_), and max (*T*_dmax_) weight loss are summarized in Table 3.

*T*_d5%_ for the PHBCL polymers increased as the PCL block length increased, such that, in PHBCL 0.5CL, *T*_d5%_ was 271 °C, whereas, in PHBCL 2.0CL, this was 290 °C. This result suggests that the PCL block acts to effectively increase the thermal stability over P3HB (for TGA curves of homopolymers see Supporting Information, Figure S20). In PHBLA, a similar trend was observed; however, *T*_d5%_ was 273 °C in PHBLA 0.5LA and 284 °C in PHBLA 2.0LA, a smaller range of values than that seen in PHBCL. In combination with the lower *T*_dmax_ values in the range of 359–365 °C in PHBLA, compared to 406–407 °C in PHBCL, the results confirm the expectation that, for the PHB-based block copolymers, the use of PCL as the partner block has a greater improvement on the thermal stability over PLA. The DTG curves for all block copolymers have two peaks; the peak arising at a lower temperature can be seen to decrease in intensity as the PCL/PLA block length increases, whereas the peak occurring at a higher temperature, roughly 360 °C and 410 °C in PHBLA and PHBCL, respectively, increases in intensity as PCL/PLA block length increases. This confirms that the first component to degrade in both the PHBCL and PHBLA diblock copolymers is PHB.

### 3.3. Crystallization Analysis

#### 3.3.1. DSC (Crystallization)

**Figure 6:**
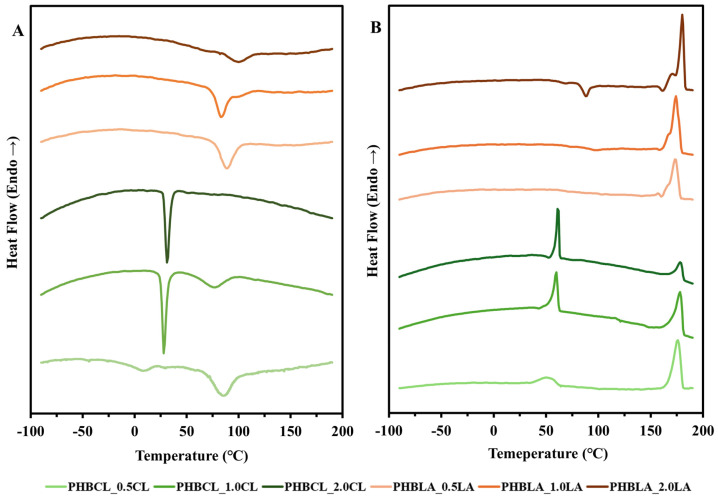
(**A**) Cooling and (**B**) second-heating DSC thermograms showing relative change in the heat flux with the temperature for the PHBCL and PHBLA diblock copolymers with varying PCL/PLA block lengths; measurements were taken in the range of −100–200 °C and at a cooling/heating rate of ±10 °C/min.

Due to the combination of the two crystalline polymers, P3HB with either PCL or PLA, an interesting study into the crystallization of crystalline–crystalline diblock copolymers was conducted by a combined analysis by DSC and POM. The DSC measurements were taken from a three-step heating regime. The samples were first heated in the range of −100–200 °C at a 20 °C/min heating rate; following a 1-min hold at 200 °C, the samples were cooled back down to −100 °C at −10 °C/min before a second heating in the range of −100–200 °C at 10 °C/min. The DSC thermograms for these runs are shown in Figure 6, with the data summarized in Table 4.

The DSC thermograms for the PHBCL diblock copolymers show a dual crystallization peak. As the PCL content increases, the crystallization of the PCL segment becomes easier, shown through the crystallization temperature (peak crystallization temperature) of the PCL segment rising from 7.3 °C in PHBCL 0.5CL to 31.2 °C in PHBCL 2.0CL. This is accompanied by the opposite trend seen for the PHB segment, where the crystallization temperature falls from 85.2 °C in PHBCL 0.5CL to 51.2 °C in PHBCL 2.0CL. Additionally, the enthalpy of fusion (Δ*H*_f_) can be seen to reduce, becoming barely detectable in PHBCL 2.0CL. These results match observations made by Liu et al., who found that, in PHBV-*b*-PCL diblock copolymers, increasing the molecular weight of the PCL segment allowed for the easier crystallization of PCL at the expense of hampering PHBV crystallization [33].

Looking at the cooling curves of the PHBLA diblock, we see that the crystallization temperature decreases from 88.5 °C in PHBLA 0.5LA to 82.6 °C in PHBLA 1.0LA, which then increases to 99.6 °C in PHBLA 2.0LA. In PHBLA 0.5LA, there is the highest weight percent ratio of PHB present; thus, PHB dominates crystallization, leading to a sharper unimodal crystallization endotherm. In PHBLA 1.0LA, a longer PLA block segment means that, not only can PLA crystallization occur, seen through the shoulder region appearing before the crystallization of PHB, but also PHB crystallization is hindered, resulting in the PHB crystallization peak occurring at a lower temperature. As the PLA block length becomes increasingly large, it becomes increasingly difficult for PHB to organize into a crystalline phase, leading to the peak seen in PHBLA 2.0LA, which is most likely a result of the crystallization of PLA, with PHB crystallization being undetectable. This would also explain the crystallization peak seen slightly in the second-heating curve of PHBLA 1.0LA, and more noticeably in PHBLA 2.0LA, wherein there would be an increasing amount of amorphous PLA that could undergo crystallization upon heating. Furthermore, the increasing *T*_m_ value for the PHBLA diblock copolymers with the increase in the PLA length, in addition to the noticeable separation and diminishing intensity of the melting peak shoulder region, can be explained by the increased presence of a PLA crystalline phase.

#### 3.3.2. POM Observation of Crystallization

**Figure 7:**
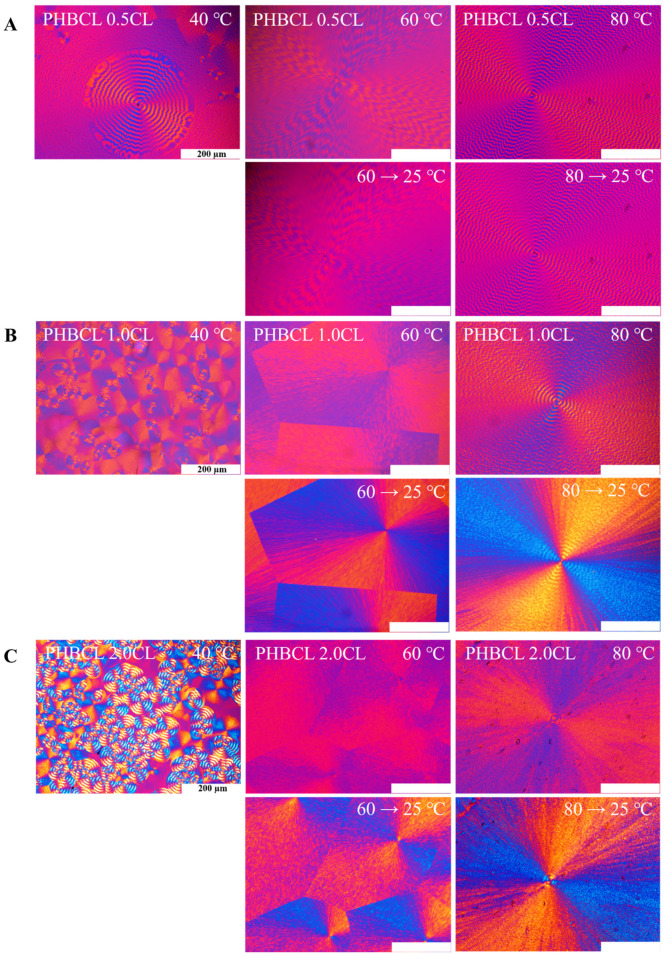
Polarized optical micrographs obtained through the isothermal crystallization of (**A**) PHBCL 0.5CL, (**B**) PHBCL 1.0CL, and (**C**) PHBCL 2.0CL. The isothermal crystallization temperatures (*T*_c_) used were 40, 60, and 80 °C. For *T*_c_ ≥ 60 °C, a sequential crystallization of PCL was permitted by rapidly cooling the hot stage to room temperature; the images obtained after the sample had reached room temperature are shown below their counterparts taken at either *T*_c_ = 60 or 80 °C. All scale bars are 200 µm.

The images obtained through the POM observation of PHBCL at *T*_c_ = 40, 60, and 80 °C are shown in Figure 7. For all compositions, we observe that, when *T*_c_ = 40 °C, two types of spherulite can be identified. When *T*_c_ = 60 and 80 °C, only a single type of spherulitic is present, although this spherulite undergoes a change in morphology upon subsequent quenching to room temperature, with the change in spherulitic morphology becoming more drastic with the increase in the PCL block length. These results come since, when *T*_c_ = 40 °C, both PHB and PCL segments are able to crystallize (coincident crystallization); however, when *T*_c_ = 60 and 80 °C, the PCL chain segment is molten, and thus, cannot crystallize. The change in birefringence direction and magnitude that occurs after quenching is a result of the crystallization of the PCL component inside the PHB negative spherulite. The fact that the change upon quenching to room temperature is barely visible in PHBCL 0.5CL at *T*_c_ = 80 °C is a result of the highly crystalline PHB preventing PCL from crystallizing. Alternatively, as the PCL block length increases, the change in birefringence that occurs upon quenching to room temperature becomes more pronounced, indicative of an increasing amount of crystallized PCL.

In Figure 7A, the PHBCL 0.5CL sample crystallized at 40 °C contains PCL as a smaller spherulite with a brighter color than the surrounding PHB. In Figure 7B, the PCL spherulite can again be identified through its spherulitic morphology being largely unchanged, with the most notable difference being in the increased number of PCL spherulites as well as their increased average radius. In Figure 7C, PHBCL 2.0CL has more PCL spherulites than was the case for PHBCL 1.0CL, but now with visible banding. A close look at the non-PCL spherulite in the PHBCL samples crystallized at 40 °C shows that the birefringence direction actually changes from being characteristic of PHB in PHBCL 0.5CL to more characteristic of the same morphology, seen when PCL crystallizes inside the PHB negative spherulite in PHBCL 1.0CL and 2.0CL. When *T*_c_ = 60 and 80 °C, the spherulite prior to quenching to room temperature can be understood to be PHB; as the PCL block length increases, this spherulite loses considerable definition, with considerable banding only observed for the PHBCL 0.5CL samples. The results thus align with the observation of the decrease in PHB crystallinity with the increase in the PCL block length observed by DSC, as well as suggesting that an increase in the PCL block length hinders the helical twisting of PHB lamella.

**Figure 8:**
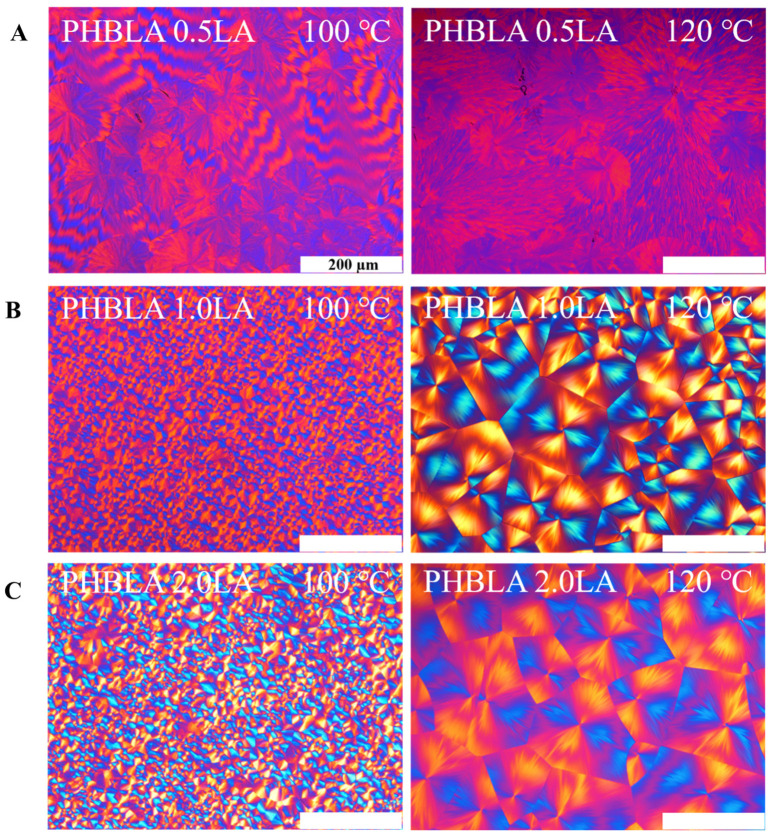
Polarized optical micrographs obtained through the isothermal crystallization of (**A**) PHBLA 0.5LA, (**B**) PHBLA 1.0LA, and (**C**) PHBLA 2.0LA of varying PLA block lengths. The isothermal crystallization temperatures (*T*_c_) used were 100 and 120 °C. All scale bars are 200 µm.

In Figure 8, polarized optical micrographs for PHBLA samples crystallized at *T*_c_ = 100 °C and 120 °C can be seen. PHBLA 1.0LA and PHBLA 2.0LA are remarkably similar in their appearance at both *T*_c_ value. At 100 °C, smaller spherulites of, at maximum, a few 10 μm in radius and less well-defined shape are present, whereas, at 120 °C, a larger spherulite is observed, suggesting a move towards a more crystalline morphology by increasing the crystallization temperature. Interestingly, the spherulites observed in PHBLA 1.0LA and PHBLA 2.0LA crystallized at 120 °C have a very weak banding structure, with most spherulites possessing a single band. The results would suggest that these spherulites are PLA crystals that possess a weak banding structure due to difficulties in helical twisting born out of being covalently bonded to a PHB segment. The decreased magnitude of birefringence (reduced color intensity) observed in PHBLA 2.0LA compared to PHBLA 1.0LA can be explained as a result of the increased presence of amorphous PLA that was also observed in the DSC curves for the analysis of crystallization. In PHBLA 0.5LA crystallized at 100 °C, banding typical of PHB can be seen to grow out of the central regions of what is most likely a very weakly crystalline PLA that contains a certain amount of PHB. This would suggest that, in PHBLA 0.5LA crystallized at 100 °C, PLA spherulites rich in PHB grow first; however, due to the lower temperature, it is more thermodynamically favorable for the PHB present in these spherulites to crystallize separately from the PLA, resulting in a final structure of highly crystalline PHB regions interspersed with considerably less crystalline PLA spherulites. When crystallized at 120 °C, PHBLA 0.5LA no longer has banding associated with crystalline PHB, with the birefringence patterns being less defined with a lower magnitude of birefringence, suggesting that the combination of a relatively high temperature and high weight fraction of PHB potentially leads to a more amorphous structure.

Overall, the POM results suggest that the crystallization of the PHBLA diblock copolymer is heavily influenced by the PLA segments’ block lengths, compared to the PHBCL diblock copolymers, whose crystallization was rather more influenced by the isothermal crystallization temperature used, i.e., at *T*_c_ < 60 °C (coincident crystallization, two-spherulite morphology) and at *T*_c_ > 60 °C (single-spherulite morphology). The observation that the AB-type diblock copolymers with two crystallizable components with very close *T*_m_ values have a crystallization dominated by the relative block lengths of the two components and those with very different *T*_m_ values are, in turn, dominated by the isothermal crystallization temperature has been made by many others [34,35,36]. Using mostly the same *T*_c_ values used in POM, melt-crystallized thin films of the PHBCL and PHBLA diblock copolymers were created and imaged using AFM.

### 3.4. AFM Observation of Surface Morphology Analysis

**Figure 9:**
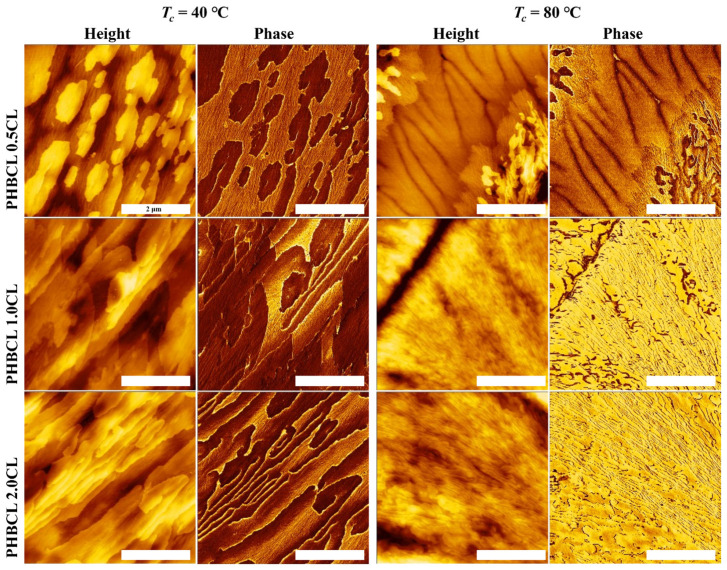
AFM height and phase images for the PHBCL diblock copolymers with varying PCL block lengths crystallized at 40 and 80 °C. All scale bars are 2 µm.

**Figure 10:**
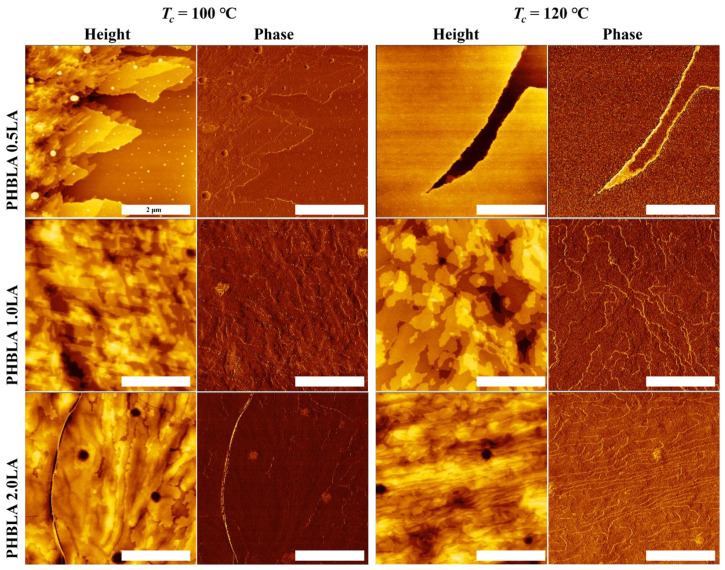
AFM height and phase images for the PHBLA diblock copolymers with varying PLA block lengths crystallized at 100 and 120 °C. All scale bars are 2 µm.

To observe the crystallized surface morphology of the PHBCL and PHBLA diblock copolymers, the thin-film samples produced by spin coating were crystallized from the melt at different isothermal crystallization temperatures (*T*_c_). For PHBCL, *T*_c_ = 40 °C and 80 °C were used, whereas, for PHBLA, *T*_c_ = 100 °C and 120 °C. AFM images for PHBCL and PHBLA are shown in Figure 9 and Figure 10, respectively. These images show a 5 × 5 µm area (scale bar = 2 µm), and additional height images with 2 × 2 µm and 20 × 20 µm areas can be found in the Supporting Information.

From Figure 9, it can be seen that the surface morphology of the PHBCL diblock copolymers changes with both an increase in the PCL block length as well as with *T*_c_. When *T*_c_ = 40 °C, PHBCL 0.5CL has a surface morphology consisting of raised islands of softer material (darker regions in AFM phase image) lying on top of a layer of harder material with a fiber-like appearance (lighter regions in AFM phase images). The observation of phase images would suggest the basal crystalline material to be PHB, whereas the raised region would be PCL, due to PHB being a harder material than PCL. Interestingly, the phase images show the raised regions of PCL to possess a border of a lighter material, suggesting PHB may also be present at the edge of these raised PCL crystalline regions. At the same *T*_c_ = 40 °C, as the PCL block length increases, the morphology of the raised spherical islands changes, creating spiral-shaped crystalline regions in PHBCL 1.0CL and PHBCL 2.0CL. The slight difference between PHBCL 1.0CL and PHBCL 2.0CL is the more confined spacing of the crystallites seen in PHBCL 2.0CL; furthermore, the phase separation in the PHBCL-type diblock copolymers appears to become less distinguishable as the PCL block length increases. These results match well with the previous results from DSC and POM that suggested a more crystalline PCL should form over PHB as the PCL block length increases.

At *T*_c_ = 80 °C, PHBCL 0.5CL has a structure similar to that at *T*_c_ = 40 °C; the fiber-like basal PHB is present with a raised PCL lying on top. However, there now appears to be a dendritic structure growing out of the basal fiber-like region. The higher crystallization temperature should allow the PHB to crystallize more steadily than was possible at *T*_c_ = 40 °C, which may explain the dendritic structure seen. The raised regions appear much smaller than when *T*_c_ = 40 °C, suggesting that the PCL region has undergone a much quicker crystallization, a result that is expected considering that the annealing treatment applied forced the very rapid crystallization of the PCL segment following the cooling down from *T*_c_ = 80 °C to room temperature. PHBCL 1.0CL and 2.0CL are defined by the disappearance of an identifiable PHB segment, with the clearly fast-growing PCL lamellar population being the only visible component. Despite this, the deep grooves that can be observed in PHBCL 1.0CL and 2.0CL (seen in Figure 9 in PHBCL 1.0CL, *T*_c_ = 80 °C, and more observable in the lower magnification images provided in the Supporting Information, Figure S10) suggest that there is an underlying shape of a very large and loosely packed dendritic structure that was controlled by the PHB that grew initially, albeit spatially hindered by the covalently bonded PCL in the molten state.

Figure 10 shows AFM height and phase images of the PHBLA diblock copolymers crystallized at *T*_c_ = 100 and 120 °C. When PHBLA 0.5LA was crystallized at *T*_c_ = 120 °C, it appears that growth typical of single-crystal PLA has occurred, with characteristic lozenge-shaped crystals and screw dislocations [23]. Certain areas of the thin-film surface in this sample have missing material, notably concentrated in screw dislocations (see Supporting Information, Figure S17); this may be a result of the fragile thin-film surface breaking at areas of higher stress upon cooling to room temperature. The phase image presented suggests that, potentially, some PHB has been relegated to the crystal edge region, with this region appearing lighter. As the PLA block length increases, morphology associated with an accelerated crystal growth rate can be observed, with increased branching and stacking of crystals. At *T*_c_ = 120 °C, PHBLA 1.0LA has a large dendritic structure; the dendrites are characterized by a long central main stem with many side branches, all possessing an angular shape. In PHBLA 2.0LA, the structure is again representative of the exaggerated rapid growth of PLA, suggesting that the PHBLA diblock copolymers crystallized at *T*_c_ = 120 °C have a crystalline morphology controlled almost entirely by the PLA block segment, with PHB potentially being contained within the PLA interlamellar regions as amorphous material.

At *T*_c_ = 100 °C, PHBLA 0.5LA has a large dendritic structure similar to that of PHBLA 1.0LA, at *T*_c_ = 120 °C; however, there are clear PHB crystalline regions overlaying the top of the dendrite that becomes noticeable towards the outer branches, shown clearly in Figure 10. This result echoes the POM observation wherein PHB crystalline regions appeared to grow out of PLA crystalline regions. PHBLA 1.0LA, at *T*_c_ = 100 °C, has a surface morphology where the PHB crystalline regions lying above the well-developed crystalline, still dendritic, PLA structure. In PHBLA 2.0LA, at *T*_c_ = 100 °C, the trend of the PHBLA diblock copolymers crystallized at *T*_c_ = 100 °C to contain both PHB and PLA crystalline regions ends, with the microstructure appearing to be fast growing PLA. These results suggest that, at *T*_c_ = 100 °C, the lower temperature permits the crystallization of PHB on top of faster crystallizing PLA in the PHBLA 0.5LA and 1.0LA samples; however, the overwhelming length of the PLA segment in PHBLA 2.0LA results in a structure wherein only crystalline PLA is observed.

### 3.5. Effects of Enzymatic Degradation on Surface Morphology

The enzymatic degradation of thin-film surfaces was undertaken by exposing the thin-film samples to PHB depolymerase, lipase, and proteinase K in separate experiments. The PHB depolymerase from *R. pickettii* T1 was used to degrade the PHB block in diblock copolymers, the lipase from *B. cepacia* was used to target PCL in PHBCL copolymers, and proteinase K from *T. album* was used to target the degradation of PLA regions in PHBLA copolymers.

**Figure 11:**
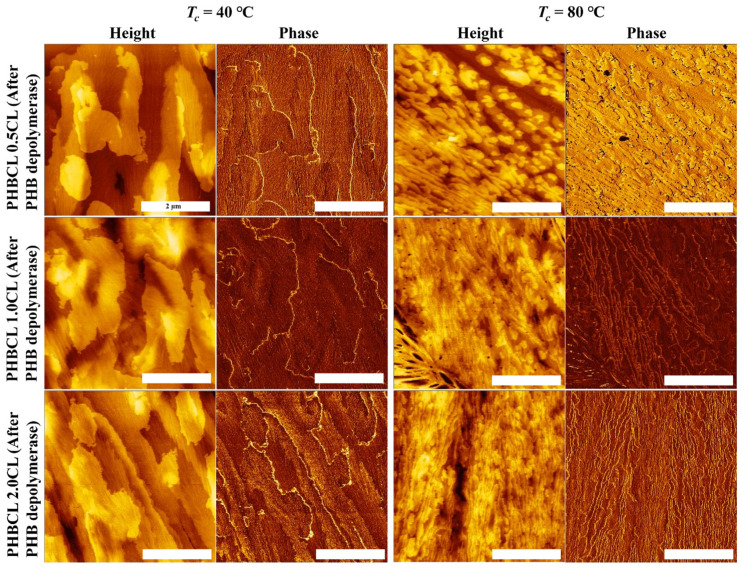
AFM height and phase images for PHBCL diblock copolymers of varying PCL block lengths crystallized at 40 and 80 °C, after 40 min of exposure to PHB depolymerase from *R. pickettii* T1. All scale bars are 2 µm.

Figure 11 shows the results of the enzymatic degradation of PHBCL diblock copolymers with PHB depolymerase. In the case of PHBCL 0.5CL, at *T*_c_ = 40 °C, it is hard to determine the effects of the PHB depolymerase; however, the higher magnification images show the fiber-like basal layer to be slightly shaved away. In PHBCL 1.0CL, at *T*_c_ = 40 °C, the degradation of the fiber-like basal layer PHB can be seen; additionally, the raised regions have undergone an observable splintering of their crystal edge regions, typical of edge-on degradation seen when PHB is targeted by PHB depolymerase [37]. Both of these observations of apparent enzymatic degradation are again exaggerated in PHBCL 2.0CL, *T*_c_ = 40 °C. These observations would suggest that the basal fiber-like region is indeed predominantly PHB, with the PHB material also being incorporated into the edges of the predominantly PCL raised regions. Considering the PHBCL copolymers at *T*_c_ = 80 °C, wherein the AFM images are more dominated by PCL crystallization, we can see that the roughening of the crystal edge regions becomes more notable as the PCL block segment length increases. This would suggest that the PHB material is indeed being preferentially incorporated into the edge of crystallized PCL regions.

**Figure 12:**
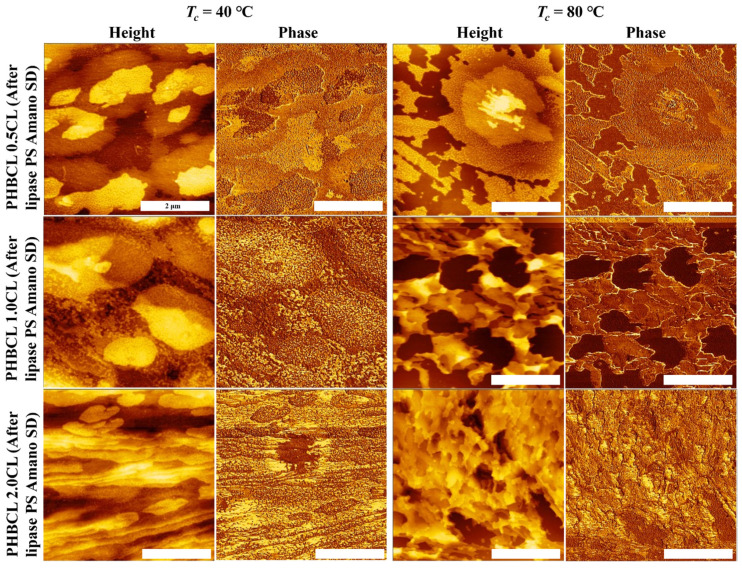
AFM height and phase images for the PHBCL diblock copolymers of varying PCL block lengths crystallized at 40 and 80 °C, after 30 min of exposure to lipase PS Amano SD from *B. cepacia*. All scale bars are 2 µm.

Figure 12 shows the effects of a lipase enzyme on the degradation of PHBCL diblock copolymers. At *T*_c_ = 40 °C, the effects of lipase are seen as an increase in the surface roughness of over the entire visible surface, including raised regions as well as certain areas of the basal region. The degradation appears to be most severe in the PHBCL 1.0CL sample, wherein the degradation of material occupying the space in between the raised regions of PCL crystallites can be clearly seen to be degraded. At *T*_c_ = 80 °C, degradation is noticeably more extreme with all samples having considerable material completely degraded. PHBCL 1.0CL and 2.0CL appear to be the most affected by the lipase enzyme; PHBCL 0.5CL appears to have retained some fiber-like regions lying below the fast-growing PCL crystal regions, which has itself been degraded such that the previously well-defined crystalline shape has been lost. Additionally, PHBCL, at *T*_c_ = 80 °C, has lost some of the previously present basal dendritic structure, suggesting that this region was mostly PCL in composition. The results of the enzymatic degradation of the PHBCL diblock copolymers crystallized at 80 °C by lipase show only fiber-like PHB to remain in the PHBCL 0.5CL sample; seeing as the other samples do not exhibit this morphology, it may be concluded that PHB crystallization is being impeded by the increasingly large PCL block to such an extent that little to no PHB crystallization is permitted, even at *T*_c_ = 80 °C, with the PCL material present being that which crystallized after the sample was removed from the hot stage and cooled to room temperature.

**Figure 13:**
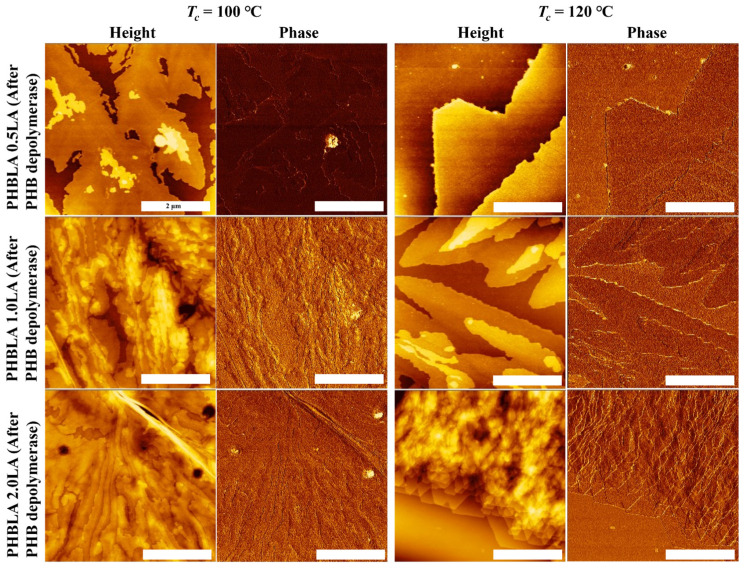
AFM height and phase images for the PHBLA diblock copolymers of varying PLA block lengths crystallized at 100 and 120 °C, after 40 min of exposure to PHB depolymerase from *R. pickettii* T1. All scale bars are 2 µm.

The PHBLA samples were targeted by PHB depolymerase, with the AFM height and phase images shown in Figure 13. In PHBLA 0.5LA, at *T*_c_ = 100 °C, the region previously associated with PHB lamellar crystals appears completely degraded, with the PLA dendritic basal layer crystal structure remaining. This drastic degradation is also seen in PHBLA, at *T*_c_ = 100 °C, where the previously clearly visible PHB lamellar crystals have been completely removed. In PHBLA 2.0LA, at *T*_c_ = 100 °C, the structure remains largely the same as it was before the addition of the PHB depolymerase enzyme. Based on the fact that, before the addition of the enzyme, the PHB crystalline regions in PHBLA diblock copolymer samples crystallized at *T*_c_ = 120 °C were hard to identify, the effects of PHB depolymerase are hard to clearly state. Despite this, in PHBLA 0.5LA, at *T*_c_ = 120 °C, the previously lightly colored region that outlined the PLA single-crystal edge has been seemingly eroded away, seemingly confirming this material at the crystal edge to be PHB. Beyond this, however, PHBLA 1.0LA and 2.0LA, at *T*_c_ = 120 °C, exhibited no marked change after 40 min exposure to PHB depolymerase, which confirms suspicions that the structures observed in the AFM images of these samples are a result of PLA crystallization.

**Figure 14:**
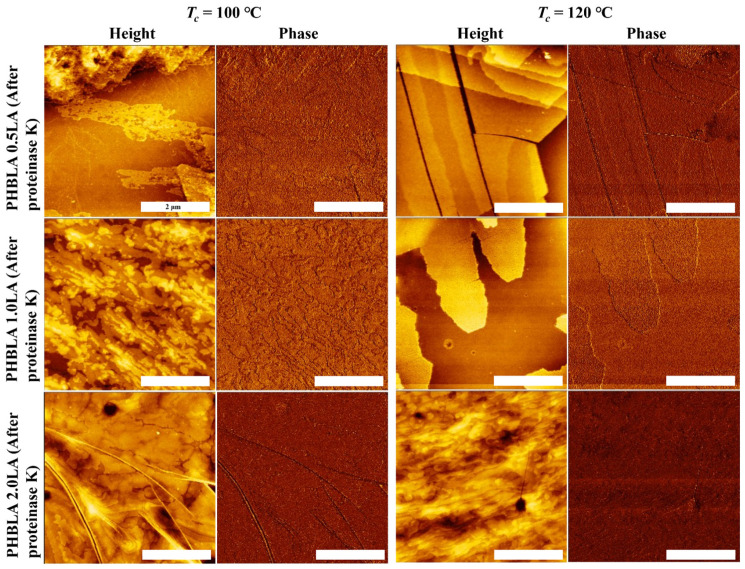
AFM height and phase images for the PHBLA diblock copolymers of varying PLA block lengths crystallized at 100 and 120 °C, after 120 min of exposure to proteinase K from *T. album*. All scale bars are 2 µm.

Figure 14 shows the AFM images obtained after exposing PHBLA samples to proteinase K for 120 min. In PHBLA 0.5LA, at *T*_c_ = 100 °C, the PHB crystalline regions were degraded to a lesser extent than when PHB depolymerase was applied. This degradation is most likely a result of the long exposure time to a slightly alkaline buffer solution as opposed to direct degradation via proteinase K. Degradation in both PHBLA 1.0LA and 2.0LA, at *T*_c_ = 100 °C, is harder to identify; in PHBLA 1.0LA, degradation again seems to have targeted the region mainly associated with PHB, with the basal PLA crystalline region only showing signs of increased surface roughness, which may suggest that the PHB crystallized over the PLA serves to protect the PLA region from enzymatic attack to a certain degree.

In the AFM images of PHBLA 0.5LA, at *T*_c_ = 120 °C, after degradation via proteinase K, deep straight-line grooves that have developed throughout the single-crystal PLA seem to have caused a fragmentation effect. On top of the fragmentation of the crystal, the lower magnification image (see Supporting Information, Figure S18) show how some areas of the crystal have been completely degraded. The slightly higher magnification images shown in Figure 14 show how crystal fragments have developed a valley-like appearance, wherein the center of the fragment seems to have been preferentially degraded over the outer sides via surface degradation. In PHBLA 1.0LA, at *T*_c_ = 120 °C, the deep fragmenting grooves are no longer present, only some shallow grooves that encroach from the crystal edge; the degradation here is mostly in the form of increased surface roughness with hollow regions appearing and generating a puckering effect homogeneously over the crystal surface. PHBLA 2.0LA, at *T*_c_ = 120 °C, exhibits a similar but less noticeable increase in surface roughness. The homogeneous increase in surface roughness is typical of PLA being degraded by proteinase K and suggests that PHBLA samples, at *T*_c_ = 120 °C, are indeed mostly crystalline PLA of varying degrees of crystallinity [38].

## 4. Conclusions

The successful chemical synthesis of PHBCL and PHBLA diblock copolymers was achieved, with characterization by ^1^H NMR and GPC used to determine the chemical structure, relative chain segment lengths, and molecular weights. A thermal analysis was completed through DSC analysis and TGA. TGA showed both the PHBCL and PHBLA diblock copolymers had an improved thermal stability over homopolymer P3HB. PHBCL and PHBLA were shown to have different crystallization regimes. PHBCL crystallization was shown to be more dependent on *T*_c_ in both the POM images and AFM surface morphologies of the PHBCL diblock copolymers. PHBLA, on the other hand, had crystallization dominated by the PHBLA PLA block length; at *T*_c_ = 100 °C, PHBLA 0.5LA and 1.0LA showed limited PHB crystalline lamellar populations, whereas, in PHBLA 2.0LA, only crystalline PLA was identified. The crystallization studies completed using DSC and POM were useful to explain, and correlated well with the surface morphology of the melt-crystallized thin films observed by AFM. The enzymatic degradation studies showed that the melt-crystallized thin films were able to be degraded by enzymes, with the degree of degradation being dependent on the surface morphology. This study has thus highlighted the impact of the composition, block length, and crystallization temperature on thin-film surface morphology and enzymatic degradation in crystalline-crystalline biodegradable block copolymers.

## Figures and Tables

**Table 1:** Conditions of the enzymatic degradation of PHB-based biodegradable diblock copolymers.

Target of Degradation	Enzyme	Enzyme (with Buffer) Solution	Volume of Enzyme Solution (μL)	Degradation Time (min)
PHB	PHB depolymerase	1.0 μg/mL in 0.1 M phosphate buffer	100	40
PCL	Lipase	1.0 mg/mL in 0.1 M phosphate buffer	75	30
PLA	Proteinase K	0.2 mg/mL in 50 mM tris-HCl buffer	100	120

**Table 2:** Structural characterization of LMPHB and PHBCL and PHBLA diblock copolymers.

Sample	PHB:PCL/PLA ^a^	*M*_n_ (g mol^−1^) ^a^	*M*_n_ (g mol^−1^) ^b^	*M*_w_ (g mol^−1^) ^b^	*M*_w_/*M*_n_^b^	Yield (%)
HMPHB	-	-	174,200	430,300	2.47	-
LMPHB	-	10,800	26,000	38,400	1.48	94
PHBCL 0.5CL	1:0.5	-	34,900	44,700	1.28	93
PHBCL 1.0CL	1:1.0	-	49,200	58,300	1.19	92
PHBCL 2.0CL	1:2.1	-	73,200	84,600	1.16	95
PHBLA 0.5LA	1:0.7	-	29,100	40,300	1.39	85
PHBLA 1.0LA	1:1.3	-	39,100	47,400	1.21	87
PHBLA 2.0LA	1:2.4	-	59,000	68,200	1.16	86

^a^ Block ratio and molecular weight measured by ^1^H NMR chemical shift integration calculations. ^b^ Molecular weight measured by GPC, with CHCl_3_ as the eluent and polystyrene standard.

**Table 3:** Thermal analysis data for the PHBCL and PHBLA diblock copolymers with varying PCL/PLA block lengths.

Sample	*T*_m_ (°C) ^a^		Δ*H*_m_ (J g^−1^) ^a^		*T*_g_ (°C) ^b^		*T*_d5%_ (°C) ^c^	*T*_d50%_ (°C) ^c^	*T*_dmax_ (°C) ^c^	*X*_c_ (%) ^d^
	PHB	PCL/ PLA + PHB	PHB	PCL/ PLA + PHB	PHB	PCL/ PLA + PHB				
PHBCL 0.5CL	166.9	47.0, 57.3	65.8	5.6, 2.0	−6.9	-	271	301	407	73.5 ± 5
PHBCL 1.0CL	168.5	58.0	56.2	40.5	−3.5	−55.0	284	378	406	65.5 ± 6
PHBCL 2.0CL	168.9	58.3	32.6	50.5	−7.8	−53.3	290	396	406	73.5 ± 5
PHBLA 0.5LA	-	165.5	-	87.4	-	10.4	273	297	359	62.5 ± 4
PHBLA 1.0LA	-	165.8	-	80.7	-	20.2	281	310	360	56.5 ± 5
PHBLA 2.0LA	-	172.3	-	67.4	-	34.5	284	346	365	56.5 ± 5

^a^ Melting temperature (*T*_m_) and enthalpy of fusion (Δ*H*_m_) were measured by DSC and taken from the first heating scan. ^b^ Glass transition temperature (*T*_g_) was measured by DSC and taken from the second heating scan. ^c^ Temperature required for 5% (*T*_d5%_), 50% (*T*_d50%_), and complete (*T*_dmax_) thermal decomposition data was measured by TGA. ^d^ Degree of crystallinity, determined from X-ray diffraction patterns.

**Table 4:** Crystallization data obtained from the DSC measurements for the PHBCL and PHBLA diblock copolymers with varying PCL/PLA block lengths.

Sample	*T*_c_ (°C) ^a^		Δ*H*_c_ (J g^−1^) ^a^		*T*_m_ (°C) ^b^		Δ*H*_f_ (J g^−1^) ^b^	
	PHB/ PHB + PLA	PCL/PLA	PHB/ PHB + PLA	PCL/PLA	PHB/ PHB + PLA	PCL/PLA	PHB/ PHB + PLA	PCL/PLA
PHBCL 0.5CL	85.2	7.3	−36.8	−9.8	176.1	51.3	55.8	20.7
PHBCL 1.0CL	77.3	28.1	−19.9	−29.5	177.9	59.8	48.6	27.8
PHBCL 2.0CL	51.2	31.2	−0.5	−41.0	178.3	61.6	28.6	33.6
PHBLA 0.5LA	88.5	-	−44.5	-	173.5	-	59.6	-
PHBLA 1.0LA	82.6	-	−35.8	-	174.0	-	59.9	-
PHBLA 2.0LA	-	99.6	-	−13.1	180.1	-	60.1	-

^a^ Crystallization temperature (*T*_c_) and enthalpy of crystallization (Δ*H*_c_) were measured by DSC and taken from the cooling scan. ^b^ Melting temperature (*T*_m_) and enthalpy of fusion (Δ*H*_m_) were measured by DSC and taken from the second-heating scan.

## Data Availability

Data are contained within the article or Supplementary Materials. The original contributions presented in this study are included in the article/Supplementary Materials. Further inquiries can be directed to the corresponding author.

## Supplementary Materials

The following supporting information can be downloaded at: https://www.mdpi.com/article/10.3390/polym17111558/s1. Figure S1. 500 MHz ^1^H NMR spectrum characterization of LMPHB in CDCl_3_, Figure S2. 500 MHz ^1^H NMR spectrum characterization of PHBCL 0.5CL in CDCl_3_, Figure S3. 500 MHz ^1^H NMR spectrum characterization of PHBCL 2.0CL in CDCl_3_, Figure S4. 500 MHz ^1^H NMR spectrum characterization of PHBLA 0.5LA in CDCl_3_, Figure S5. 500 MHz ^1^H NMR spectrum characterization of PHBLA 2.0LA in CDCl_3_, Table S1. Interplanar *d*-Spacings (Å) obtained from X-ray diffraction analysis for PHBCL and PHBLA diblock copolymers with varying PCL/PLA block lengths, Table S2. Weight % of PHB calculated theoretical values against values as measured by TGA, Figure S6. Polarized optical micrograph showing a large ring-banded spherulite of LMPHB, obtained through isothermal crystallization of LMPHB at *T*_c_ = 80 °C. The scale bar is 200 μm, Figure S7. AFM height images for PHBCL diblock copolymers with varying PCL block lengths crystallized at 40 °C, Figure S8. AFM height images for PHBCL diblock copolymers with varying PCL block lengths crystallized at 40 °C after 40 min exposure to PHB depolymerase from *R. picketti* T1, Figure S9. AFM height images for PHBCL diblock copolymers with varying PCL block lengths crystallized at 40 °C after 30 min exposure to lipase PS amano SD from *B. cepacia*, Figure S10. AFM height images for PHBCL diblock copolymers with varying PCL block lengths crystallized at 80 °C, Figure S11. AFM height images for PHBCL diblock copolymers with varying PCL block lengths crystallized at 80 °C after 40 min exposure to PHB depolymerase from *R. picketti* T1, Figure S12. AFM height images for PHBCL diblock copolymers with varying PCL block lengths crystallized at 80 ℃ after 30 min exposure to lipase PS amano SD from *B. cepacia*, Figure S13. AFM height images for PHBLA diblock copolymers with varying PLA block lengths crystallized at 100 °C, Figure S14. AFM height images for PHBLA diblock copolymers with varying PLA block lengths crystallized at 100 °C after 40 min exposure to PHB depolymerase from *R. picketti* T1, Figure S15. AFM height images for PHBLA diblock copolymers with varying PLA block lengths crystallized at 100 ℃ after 120 min exposure to proteinase K from *T. album*, Figure S16. AFM height images for PHBLA diblock copolymers with varying PLA block lengths crystallized at 120 ℃, Figure S17. AFM height images for PHBLA diblock copolymers with varying PLA block lengths crystallized at 120 °C after 40 min exposure to PHB depolymerase from *R. picketti* T1, Figure S18. AFM height images for PHBLA diblock copolymers with varying PLA block lengths crystallized at 100 ℃ after 120 min exposure to proteinase K from *T. album*, Figure S19. DSC 2nd heating thermograms enlarged to highlight the shifting *T*_g_ exhibited by PHBLA diblock copolymers, Figure S20. TGA curves for PHB, PCL and PLA homopolymers between 40–500 °C using a 10 °C/min heating regime, Figure S21. (A) 1st heating and (B) 2nd heating DSC thermograms showing relative change in heat flux with temperature for PHB, PCL and PLA homopolymers, Table S3. Summary of thermal analysis of PHB, PCL and PLA homopolymers.

## Abbreviations

The following abbreviations are used in this manuscript:

P3HB (or PHB)	Poly[(*R*)-3-hydroxybutyrate]
PCL	Polycaprolactone
PLLA (or PLA)	Poly(l-lactic acid)
LMPHB	Low molecular weight poly[(*R*)-3-hydroxybutyrate]
HMPHB	High molecular weight poly[(*R*)-3-hydroxybutyrate]
PHBCL	Poly[(*R*)-3-hydroxybutyrate]-*block*-polycaprolactone
PHBLA	Poly[(*R*)-3-hydroxybutyrate]-*block*-polylactic acid
NMR	Nuclear magnetic resonance
GPC	Gas permeation chromatography
DSC	Differential scanning calorimetry
TGA	Thermal gravimetric analysis
DTG	Derivative thermogravimetric analysis
WAXD	Wide-angle X-ray diffraction
POM	Polarized optical microscopy
AFM	Atomic force microscopy
ROP	Ring- opening polymerization
DBTDL	Dibutyltin dilaurate

## References

[1] Swetha T.A., Bora A., Ananthy V., Ponnuchamy K., Muthusamy G., Arun A. (2024). A review of bioplastics as an alternative to petrochemical plastics: Its types, structure, characteristics, degradation, standards, and feedstocks. Polym. Adv. Technol..

[2] Di Bartolo A., Infurna G., Dintcheva N.T. (2021). A Review of Bioplastics and Their Adoption in the Circular Economy. Polymers.

[3] Cakmak O.K. (2024). Biodegradable Polymers—A Review on Properties, Processing, and Degradation Mechanism. Circ. Econ. Sustain..

[4] Asghari F., Samiei M., Adibkia K., Akbarzadeh A., Davaran S. (2017). Biodegradable and biocompatible polymers for tissue engineering application: A review. Artif. Cells Nanomed. Biotechnol..

[5] Meereboer K.W., Misra M., Mohanty A.K. (2020). Review of recent advances in the biodegradability of polyhydroxyalkanoate (PHA) bioplastics and their composites. Green Chem..

[6] Samir A., Ashour F.H., Hakim A.A.A., Bassyouni M. (2022). Recent advances in biodegradable polymers for sustainable applications. Npj Mater. Degrad..

[7] Rai P., Mehrotra S., Priya S., Gnansounou E., Sharma S.K. (2021). Recent advances in the sustainable design and applications of biodegradable polymers. Bioresour. Technol..

[8] Kai D., Loh X.J. (2014). Polyhydroxyalkanoates: Chemical Modifications Toward Biomedical Applications. ACS Sustain. Chem. Eng..

[9] Kumar N., Ravikumar M.N.V., Domb A.J. (2001). Biodegradable block copolymers. Adv. Drug Deliv. Rev..

[10] Reeve M.S., McCarthy S.P., Gross R.A. (1993). Preparation and characterization of (R)-poly(.beta.-hydroxybutyrate)-poly(.epsilon.-caprolactone) and (R)-poly(.beta.-hydroxybutyrate)-poly(lactide) degradable diblock copolymers. Macromolecules.

[11] Oyama T., Kobayashi S., Okura T., Sato S., Tajima K., Isono T., Satoh T. (2019). Biodegradable Compatibilizers for Poly(hydroxyalkanoate)/Poly(ε-caprolactone) Blends through Click Reactions with End-Functionalized Microbial Poly(hydroxyalkanoate)s. ACS Sustain. Chem. Eng..

[12] Jeepery I.F., Goto T., Sudesh K., Abe H. (2023). Biodegradable block copolymer as compatibilizer and blend component of poly(3-hydroxybutyrate-co-3-hydroxyhexanoate)-based polyester blends. Eur. Polym. J..

[13] Wu L., Chen S., Li Z., Xu K., Chen G.-Q. (2008). Synthesis, characterization and biocompatibility of novel biodegradable poly[((R)-3-hydroxybutyrate)-block-(D,L-lactide)-block-(ε-caprolactone)] triblock copolymers. Polym. Int..

[14] Wu L., Wang L., Wang X., Xu K. (2010). Synthesis, characterizations and biocompatibility of novel biodegradable star block copolymers based on poly[(R)-3-hydroxybutyrate] and poly(ε-caprolactone). Acta Biomater..

[15] Handbook of Polymer Crystallization. https://onlinelibrary.wiley.com/doi/epdf/10.1002/9781118541838.

[16] Hamley I.W., Castelletto V., Castillo R.V., Müller A.J., Martin C.M., Pollet E., Dubois P. (2005). Crystallization in Poly(l-lactide)-b-poly(ε-caprolactone) Double Crystalline Diblock Copolymers: A Study Using X-ray Scattering, Differential Scanning Calorimetry, and Polarized Optical Microscopy. Macromolecules.

[17] Li S., Sun X., Li H., Yan S. (2018). The crystallization behavior of biodegradable polymer in thin film. Eur. Polym. J..

[18] Tsuji H., Miyauchi S. (2001). Poly(l-lactide): VI Effects of crystallinity on enzymatic hydrolysis of poly(l-lactide) without free amorphous region. Polym. Degrad. Stab..

[19] Reeve M.S., McCarthy S.P., Downey M.J., Gross R.A. (1994). Polylactide stereochemistry: Effect on enzymic degradability. Macromolecules.

[20] Kikkawa Y., Abe H., Iwata T., Inoue Y., Doi Y. (2002). Crystallization, Stability, and Enzymatic Degradation of Poly(l-lactide) Thin Film. Biomacromolecules.

[21] Abe H., Doi Y., Aoki H., Akehata T., Hori Y., Yamaguchi A. (1995). Physical Properties and Enzymic Degradability of Copolymers of (R)-3-Hydroxybutyric and 6-Hydroxyhexanoic Acids. Macromolecules.

[22] Ghosh K., Jones B.H. (2021). Roadmap to Biodegradable Plastics—Current State and Research Needs. ACS Sustain. Chem. Eng..

[23] Iwata T., Doi Y. (1998). Morphology and Enzymatic Degradation of Poly(l-lactic acid) Single Crystals. Macromolecules.

[24] Numata K., Abe H., Doi Y. (2008). Enzymatic processes for biodegradation of poly(hydroxyalkanoate)s crystals. Can. J. Chem..

[25] Vonk C.G. (1973). Computerization of Ruland’s X-ray method for determination of the crystallinity in polymers. J. Appl. Crystallogr..

[26] Tan L.-T., Hiraishi T., Sudesh K., Maeda M. (2013). Directed evolution of poly[(R)-3-hydroxybutyrate] depolymerase using cell surface display system: Functional importance of asparagine at position 285. Appl. Microbiol. Biotechnol..

[27] Špitalský Z., Lacík I., Lathová E., Janigová I., Chodák I. (2006). Controlled degradation of polyhydroxybutyrate via alcoholysis with ethylene glycol or glycerol. Polym. Degrad. Stab..

[28] Li Y., Kissel T. (1998). Synthesis, characteristics and in vitro degradation of star-block copolymers consisting of l-lactide, glycolide and branched multi-arm poly(ethylene oxide). Polymer.

[29] Timbart L., Renard E., Tessier M., Langlois V. (2007). Monohydroxylated Poly(3-hydroxyoctanoate) Oligomers and Its Functionalized Derivatives Used as Macroinitiators in the Synthesis of Degradable Diblock Copolyesters. Biomacromolecules.

[30] Makiguchi K., Satoh T., Kakuchi T. (2011). Diphenyl Phosphate as an Efficient Cationic Organocatalyst for Controlled/Living Ring-Opening Polymerization of δ-Valerolactone and ε-Caprolactone. Macromolecules.

[31] Kim M.S., Seo K.S., Khang G., Lee H.B. (2005). Ring-Opening Polymerization of *ε*-Caprolactone by Poly(ethylene glycol) by an Activated Monomer Mechanism. Macromol. Rapid Commun..

[32] Castillo R., Müller A. (2009). Crystallization and morphology of biodegradable or biostable single and double crystalline block copolymers. Prog. Polym. Sci..

[33] Liu Q., Shyr T.-W., Tung C.-H., Deng B., Zhu M. (2011). Block copolymers containing poly (3-hydroxybutyrate-co-3-hydroxyvalerate) and poly (ɛ-caprolactone) units: Synthesis, characterization and thermal degradation. Fibers Polym..

[34] He C., Sun J., Deng C., Zhao T., Deng M., Chen X., Jing X. (2004). Study of the Synthesis, Crystallization, and Morphology of Poly(ethylene glycol)−Poly(ε-caprolactone) Diblock Copolymers. Biomacromolecules.

[35] Van Horn R.M., Zheng J.X., Sun H.-J., Hsiao M.-S., Zhang W.-B., Dong X.-H., Xu J., Thomas E.L., Lotz B., Cheng S.Z.D. (2010). Solution Crystallization Behavior of Crystalline−Crystalline Diblock Copolymers of Poly(ethylene oxide)-*block*-poly(ε-caprolactone). Macromolecules.

[36] Xu Y., Zhang Y., Fan Z., Li S. (2010). Melt crystallization and morphology of poly(Îµ-caprolactone)-poly(ethylene glycol) diblock copolymers with different compositions and molecular weights. J. Polym. Sci. Part B Polym. Phys..

[37] Iwata T., Doi Y., Kasuya K.-I., Inoue Y. (1997). Visualization of Enzymatic Degradation of Poly[(*R*)-3-hydroxybutyrate] Single Crystals by an Extracellular PHB Depolymerase. Macromolecules.

[38] Kurokawa K., Yamashita K., Doi Y., Abe H. (2006). Surface properties and enzymatic degradation of end-capped poly(l-lactide). Polym. Degrad. Stab..

