# Supplementary material for: Surface Morphology and Degradation of Poly[(R)-3-Hydroxybutyrate]-block-Poly(ε-Caprolactone) and Poly[(R)-3-Hydroxybutyrate]-block-Poly(l-Lactide) Biodegradable Diblock Copolymers
Source: Polymers (Basel). 2025 Jun 3;17(11):1558. doi: 10.3390/polym17111558 (PMC12157110; doi:10.3390/polym17111558)
Supplement: Supplementary file 1 [file polymers-17-01558-s001.zip › polymers-3678199-supplementary.pdf]

Supporting Information

Surface Morphology and Degradation of Poly[(*R*)-3-Hydroxybutyrate]-*block*-Poly(ε-Caprolactone) and Poly[(*R*)-3-Hydroxybutyrate]-*block*-Poly(L-Lactide) Biodegradable Diblock Copolymers.

Ayan Bartels-Ellis [a, b], Senri Hayashi [a], Tomohiro Hiraishi [a], Takeharu Tsuge [b], Hideki Abe [a] *

[a] Bioplastic Research Team, RIKEN Center for Sustainable Resource Science, 2-1 Hirosawa, Wako, Saitama 351-0198, Japan

[b] Department of Materials Science and Engineering, Institute of Science Tokyo, 4259 Nagatsuta, Midori-ku, Yokohama, 226-8502, Japan

Figure and Tables Caption

Figure S1. 500 MHz ^{1}H NMR spectrum characterization of LMPHB in $CDCl_3$.

Figure S2. 500 MHz ^{1}H NMR spectrum characterization of PHBCL 0.5CL in $CDCl_3$.

Figure S3. 500 MHz ^{1}H NMR spectrum characterization of PHBCL 2.0CL in $CDCl_3$.

Figure S4. 500 MHz ^{1}H NMR spectrum characterization of PHBLA 0.5LA in $CDCl_3$.

Figure S5. 500 MHz ^{1}H NMR spectrum characterization of PHBLA 2.0LA in $CDCl_3$.

Table S1. Interplanar *d*-Spacings (Å) obtained from X-ray diffraction analysis for PHBCL and PHBLA diblock copolymers with varying PCL/PLA block lengths.

Table S2. Weight % of PHB calculated theoretical values against values as measured by TGA.

Figure S6. Polarized optical micrograph showing a large ring-banded spherulite of LMPHB, obtained through isothermal crystallization of LMPHB at T_c = 80 °C. The scale bar is 200 μm.

Figure S7. AFM height images for PHBCL diblock copolymers with varying PCL block lengths crystallized at 40 °C.

Figure S8. AFM height images for PHBCL diblock copolymers with varying PCL block lengths crystallized at 40 °C after 40 minutes exposure to PHB depolymerase from *R. picketti* T1.

Figure S9. AFM height images for PHBCL diblock copolymers with varying PCL block lengths crystallized at 40 °C after 30 minutes exposure to lipase PS amano SD from *B. cepacia*.

Figure S10. AFM height images for PHBCL diblock copolymers with varying PCL block lengths crystallized at 80 °C.

Figure S11. AFM height images for PHBCL diblock copolymers with varying PCL block lengths crystallized at 80 °C after 40 minutes exposure to PHB depolymerase from *R. picketti* T1.

Figure S12. AFM height images for PHBCL diblock copolymers with varying PCL block lengths crystallized at 80 °C after 30 minutes exposure to lipase PS amano SD from *B. cepacia*.

Figure S13. AFM height images for PHBLA diblock copolymers with varying PLA block lengths crystallized at 100 °C.

Figure S14. AFM height images for PHBLA diblock copolymers with varying PLA block lengths crystallized at 100 °C after 40 minutes exposure to PHB depolymerase from *R. picketti* T1.

Figure S15. AFM height images for PHBLA diblock copolymers with varying PLA block lengths crystallized at 100 °C after 120 minutes exposure to proteinase K from *T. album*.

Figure S16. AFM height images for PHBLA diblock copolymers with varying PLA block lengths crystallized at 120 °C.

Figure S17. AFM height images for PHBLA diblock copolymers with varying PLA block lengths crystallized at 120 °C after 40 minutes exposure to PHB depolymerase from *R. picketti* T1.

Figure S18. AFM height images for PHBLA diblock copolymers with varying PLA block lengths crystallized at 100 °C after 120 minutes exposure to proteinase K from *T. album*.

Figure S19. DSC 2nd heating thermograms enlarged to highlight the shifting T_g exhibited by PHBLA diblock copolymers.

Figure S20. TGA curves for PHB, PCL and PLA homopolymers between 40 - 500°C using a 10°C/min heating regime.

Figure S21. (A) 1st heating and **(B)** 2nd heating DSC thermograms showing relative change in heat flux with temperature for PHB, PCL and PLA homopolymers.

Table S3. Summary of thermal analysis of PHB, PCL and PLA homopolymers.

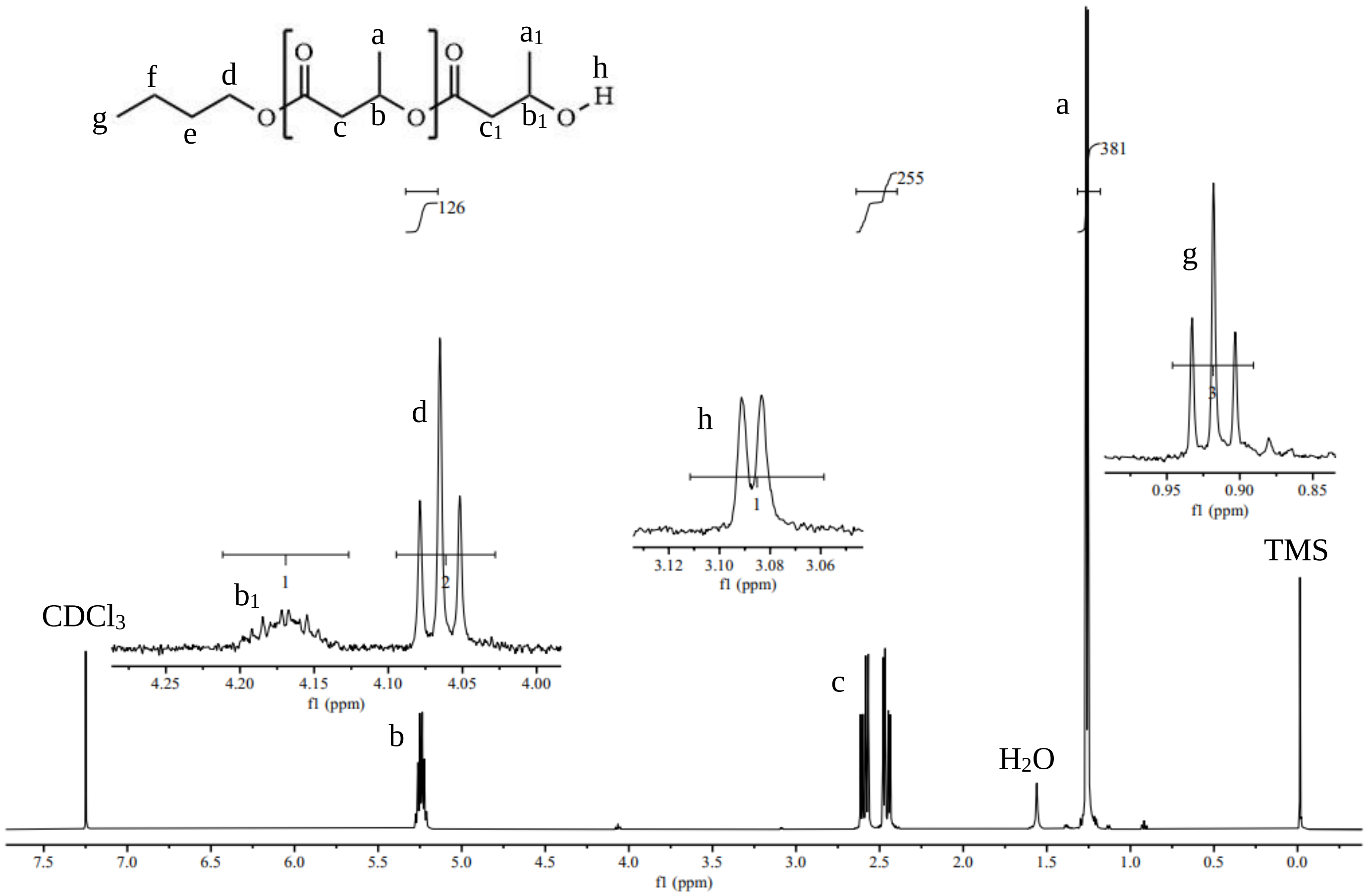

Figure S1. 500 MHz ^{1}H NMR spectrum characterization of LMPHB in $CDCl_3$.

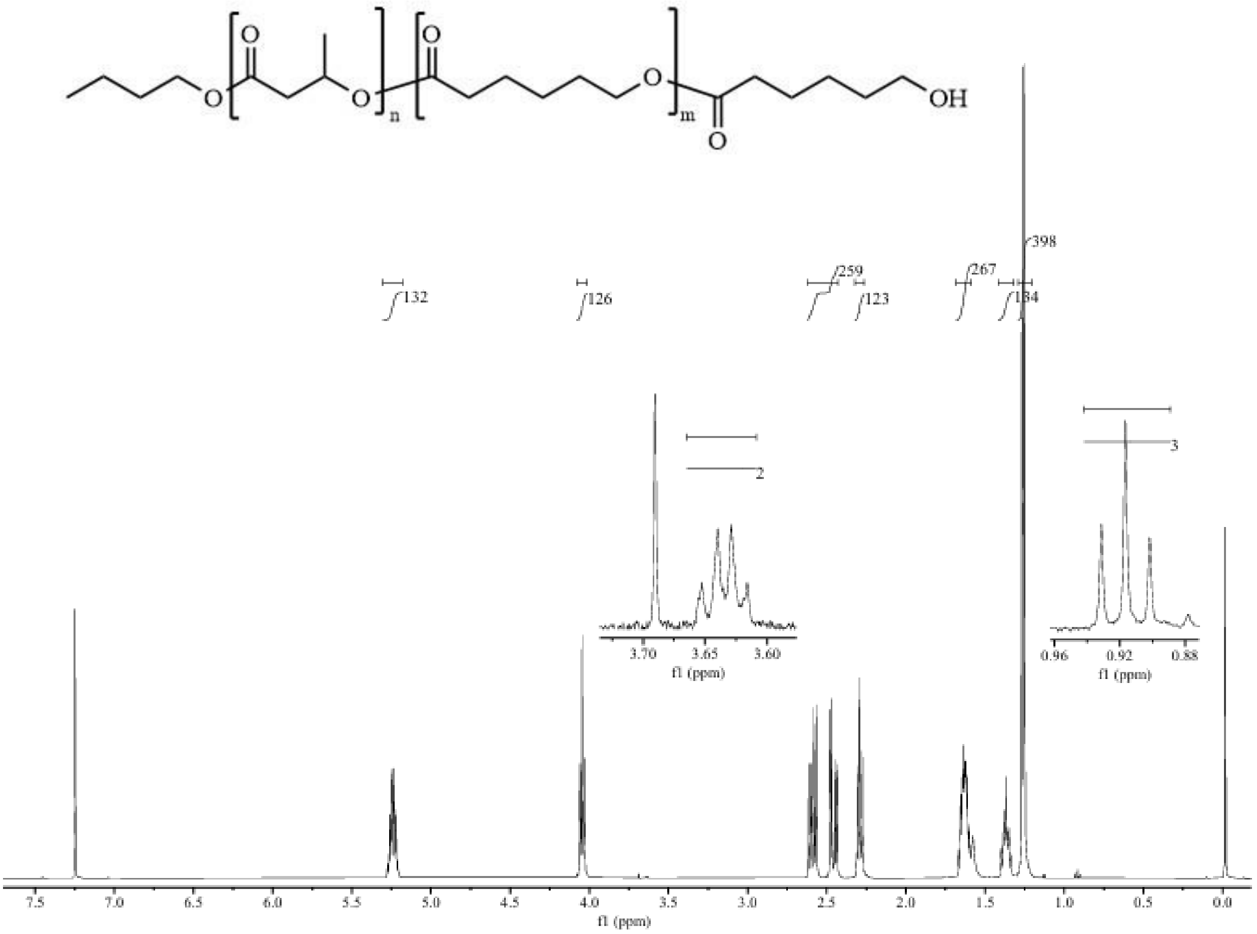

Figure S2. 500 MHz ^{1}H NMR spectrum characterization of PHBCL 0.5CL in $CDCl_3$.

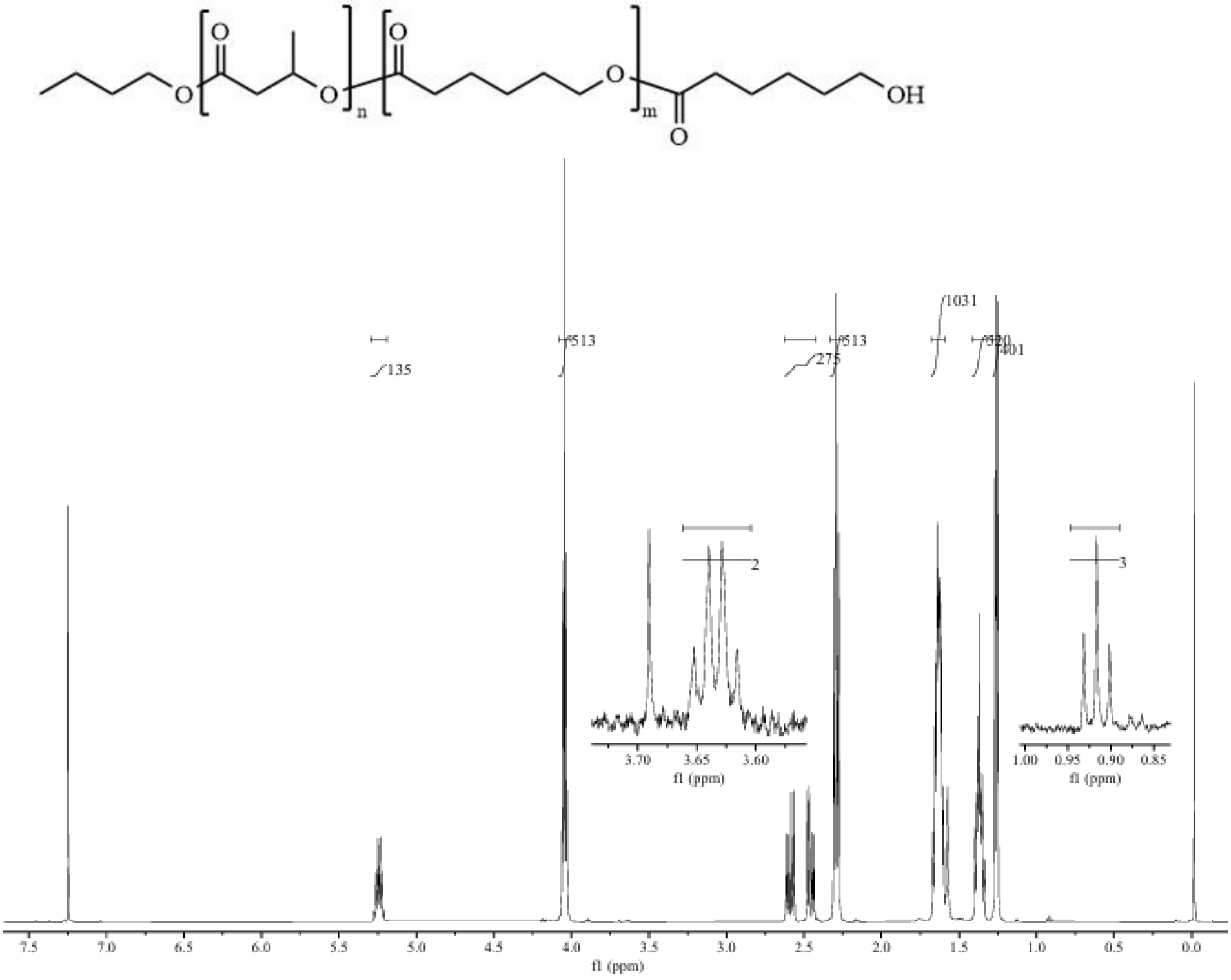

Figure S3. 500 MHz ¹H NMR spectrum characterization of PHBCL 2.0CL in $CDCl_3$.

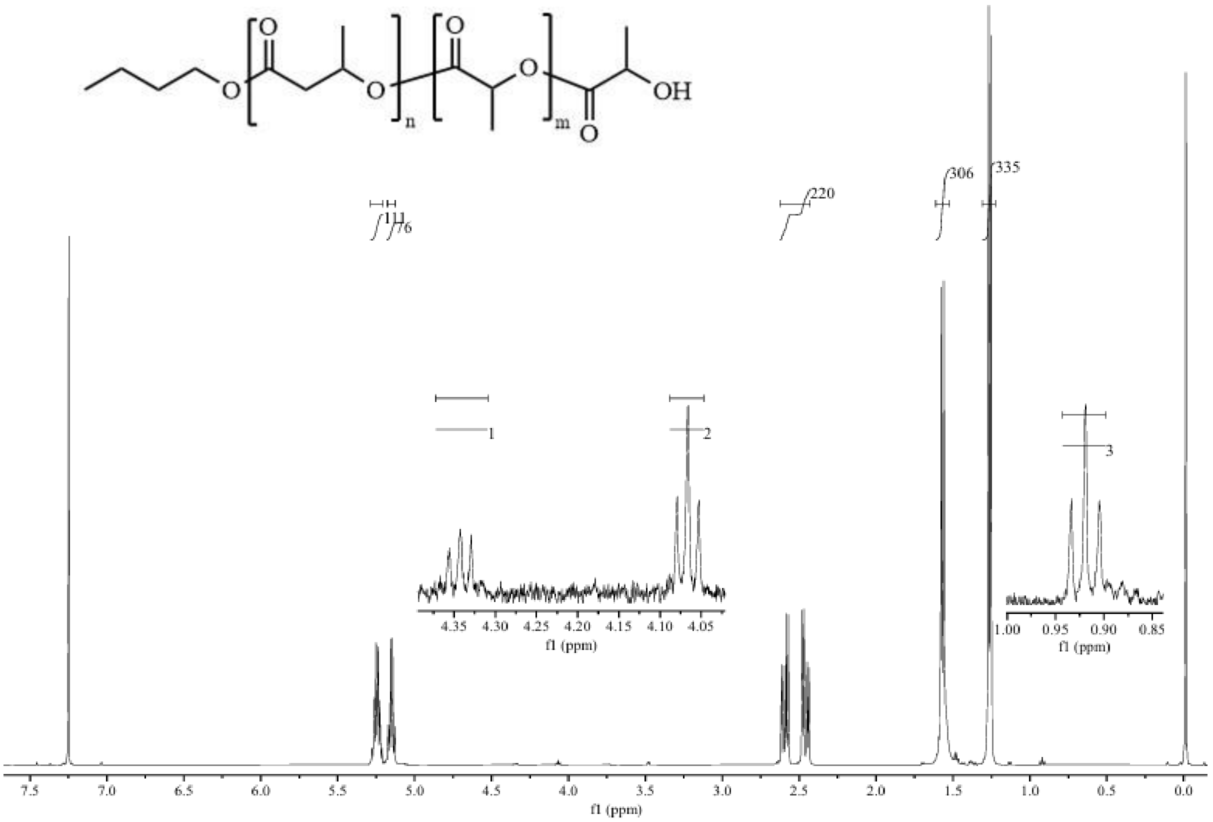

Figure S4. 500 MHz ^{1}H NMR spectrum characterization of PHBLA 0.5LA in $CDCl_3$.

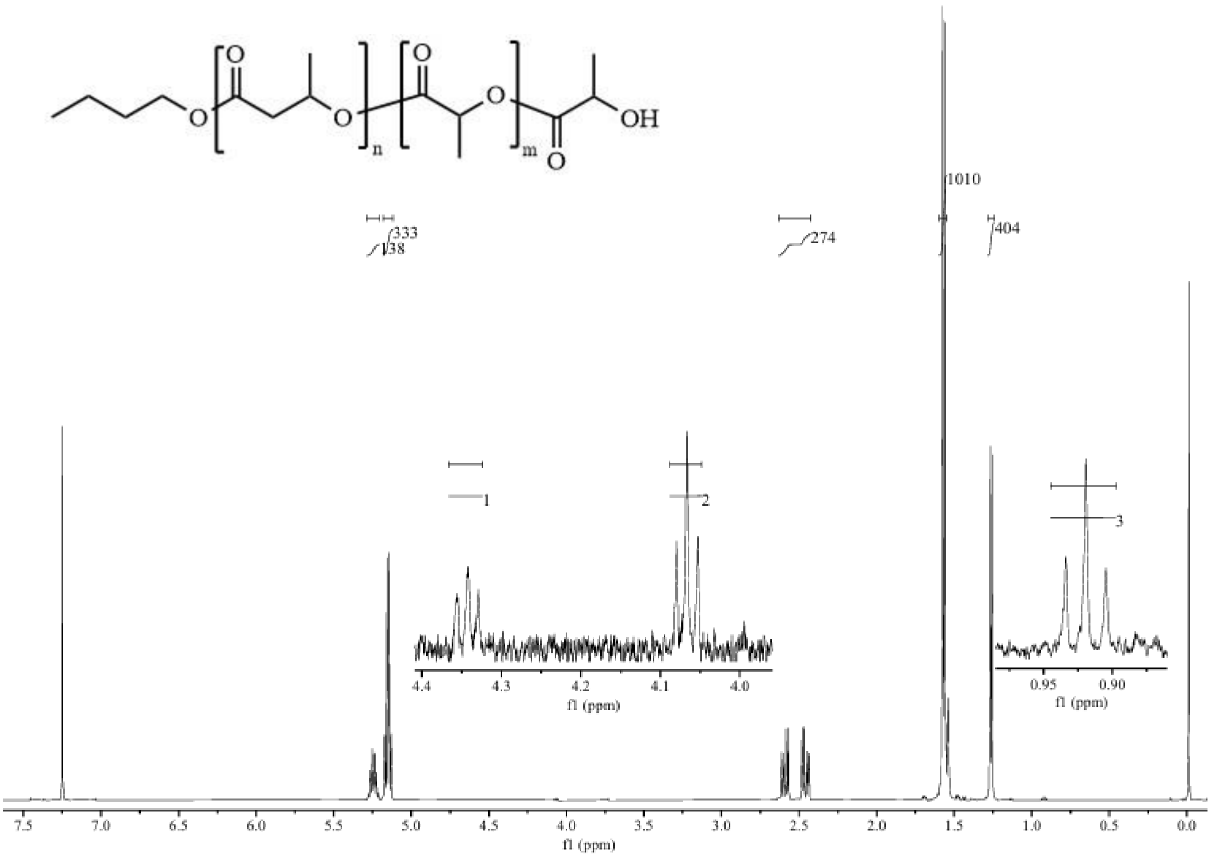

Figure S5. 500 MHz ^{1}H NMR spectrum characterization of PHBLA 2.0LA in $CDCl_3$.

Table S1. Interplanar *d*-Spacings (Å) obtained from X-ray diffraction analysis for PHBCL and PHBLA diblock copolymers with varying PCL/PLA block lengths.

PHBCL 0.5CL	PHBCL 1.0CL	PHBCL 2.0CL	PHBLA 0.5LA	PHBLA 1.0LA	PHBLA 2.0LA
6.52	6.52	6.49	6.48	6.51	6.49
5.19	5.18	5.17	5.26	5.25	5.26
4.12	4.13	4.12	4.62		4.60
3.76	3.74	3.72	3.95	4.06	3.97
3.48	3.46	3.45	3.47	3.48	3.48
3.27	3.27				
2.94	2.94	2.94	2.91	2.93	2.87

Table S2. Weight % of PHB calculated theoretical values against values as measured by TGA.

Sample	Weight % PHB [a]	Weight % PHB [b]
PHBCL 0.5CL	60	60
PHBCL 1.0CL	46	45
PHBCL 2.0CL	28	28
PHBLA 0.5LA	71	71
PHBLA 1.0LA	55	53
PHBLA 2.0LA	35	35

[a] Theoretical values of weight % as calculated from ^{1}H NMR PHB : PCL molar ratio.

[b] Values of weight % as measured by the first stage change in weight % in TGA curves.

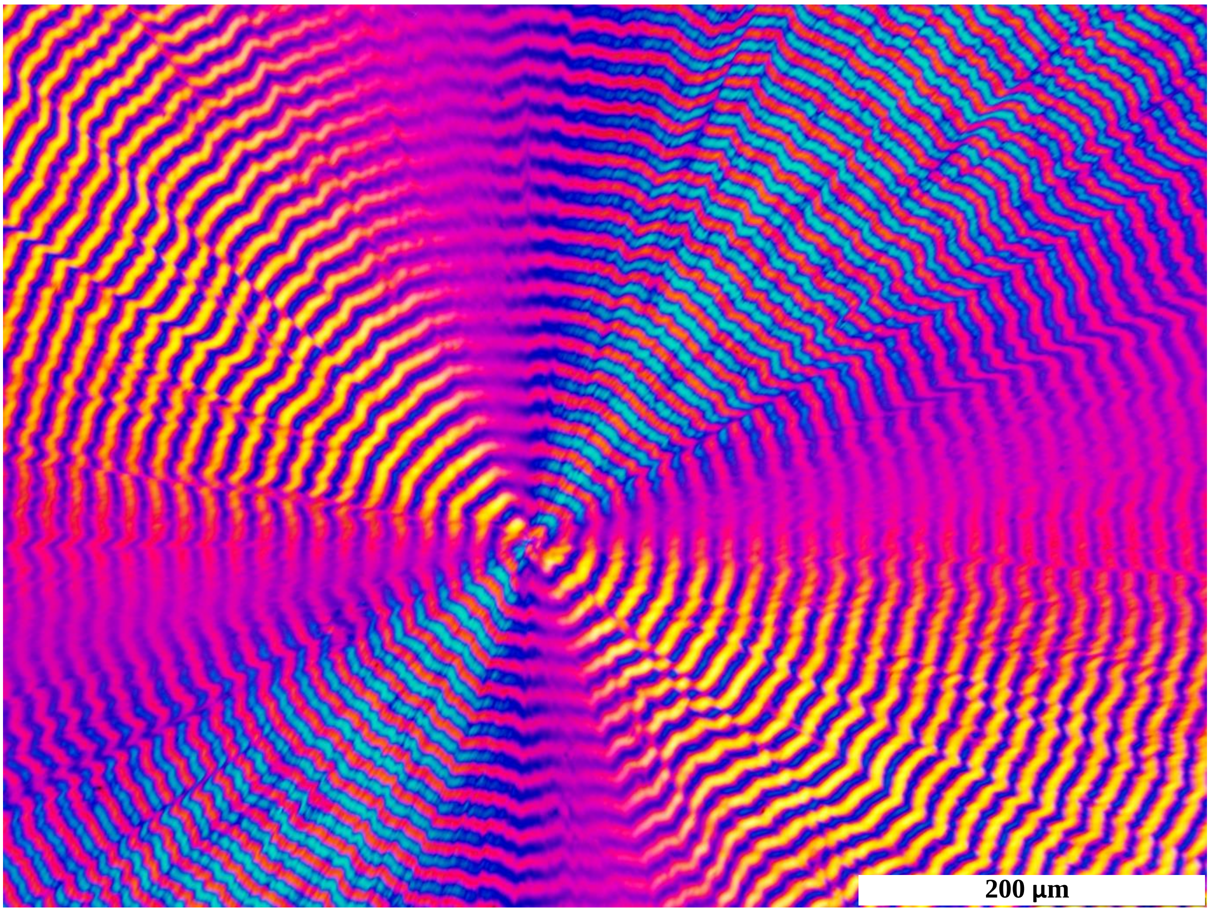

Figure S6. Polarized optical micrograph showing a large ring-banded spherulite of LMPHB, obtained through isothermal crystallization of LMPHB at T_c = 80 °C. The scale bar is 200 μm.

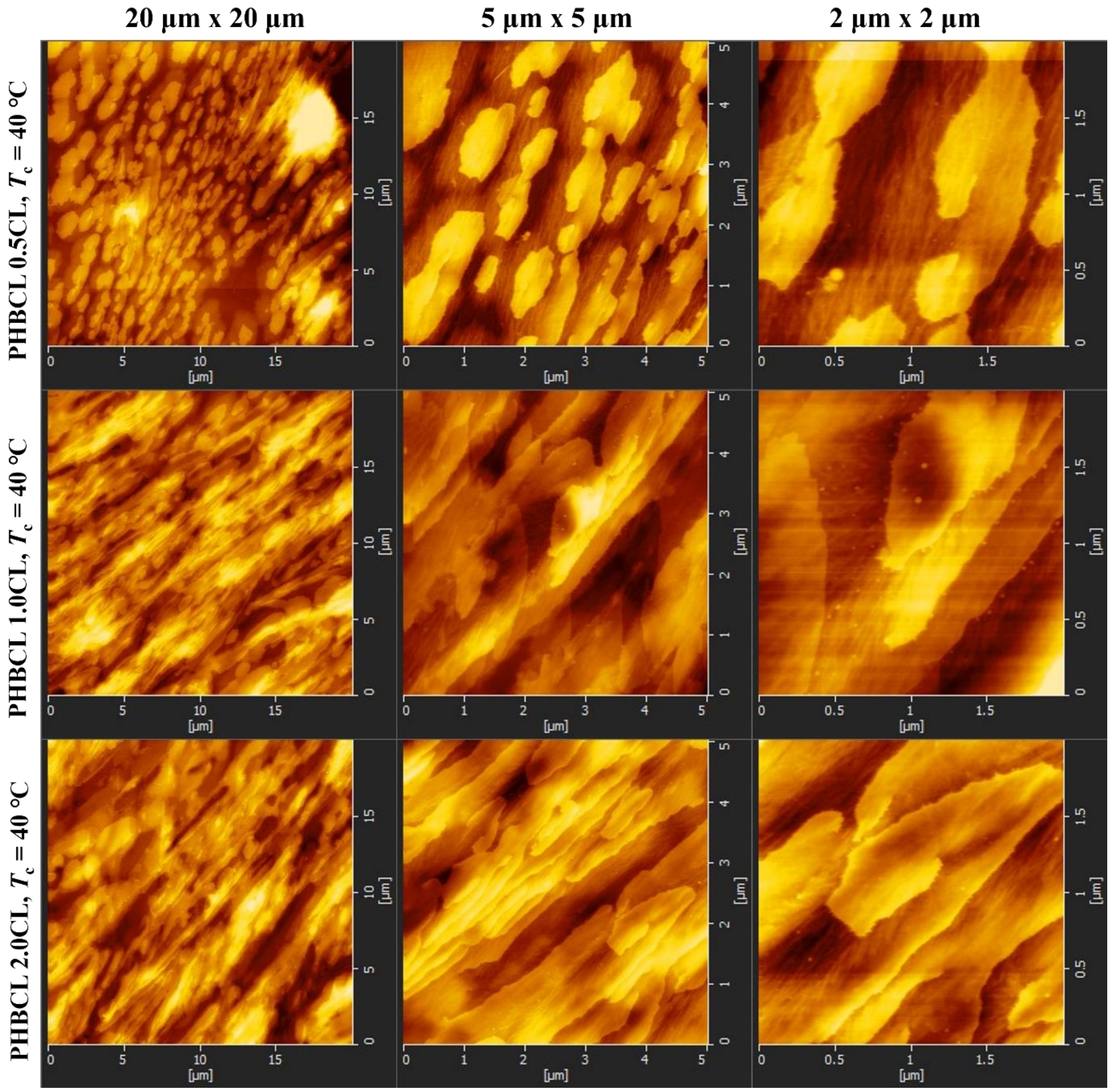

Figure S7. AFM height images for PHBCL diblock copolymers with varying PCL block lengths crystallized at 40 °C.

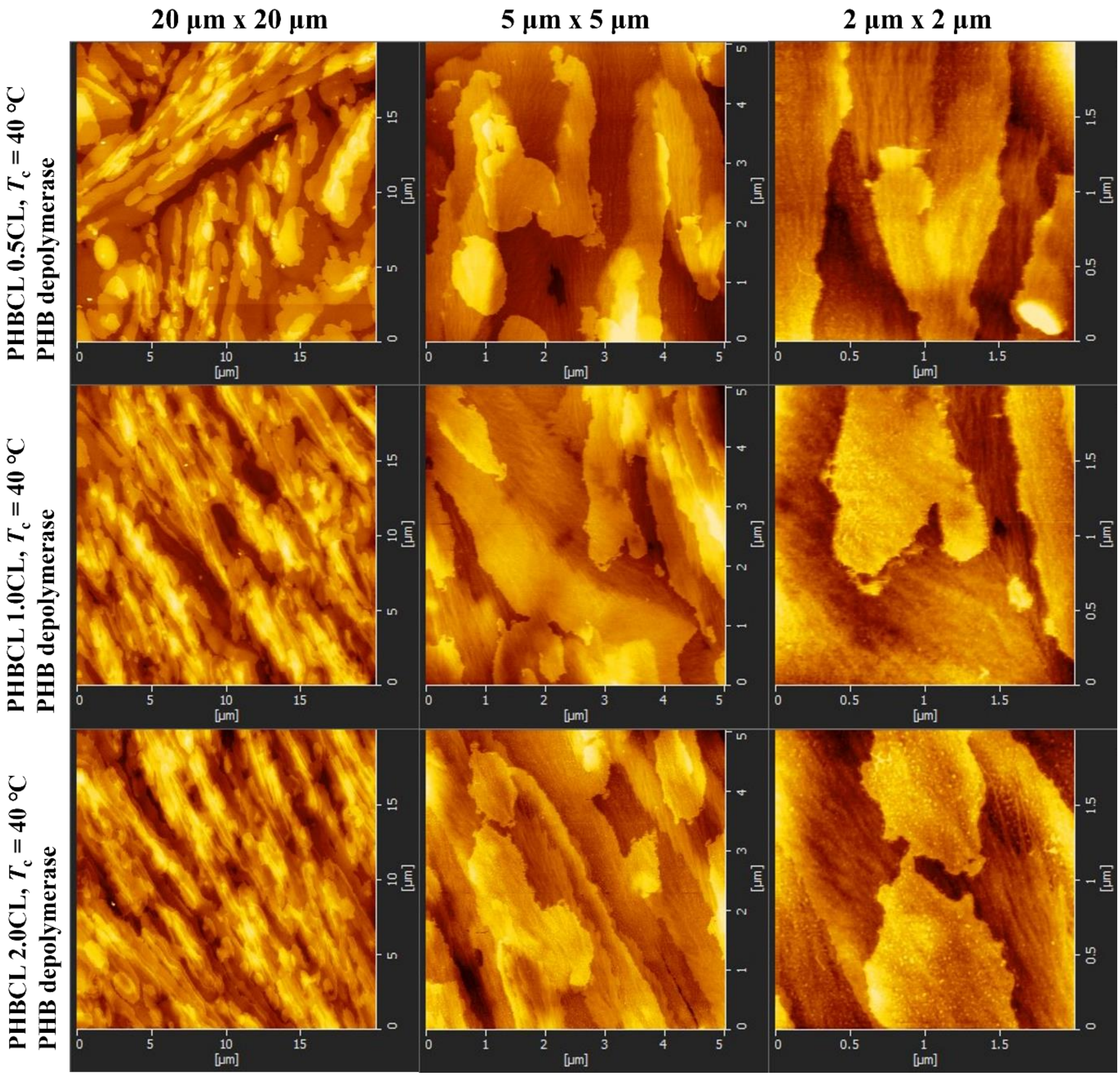

Figure S8. AFM height images for PHBCL diblock copolymers with varying PCL block lengths crystallized at 40 °C after 40 minutes exposure to PHB depolymerase from *R. picketti* T1.

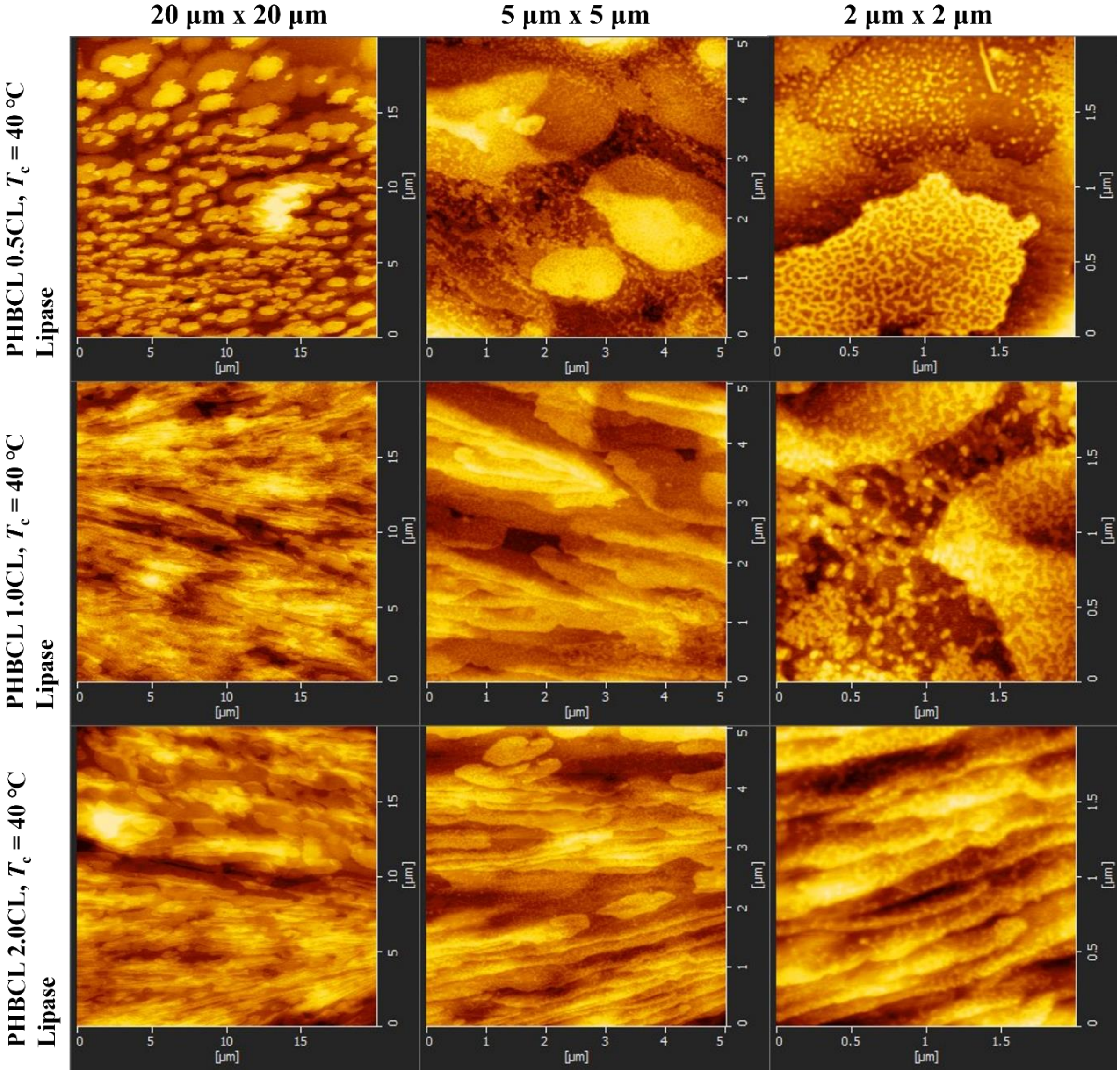

Figure S9. AFM height images for PHBCL diblock copolymers with varying PCL block lengths crystallized at 40 °C after 30 minutes exposure to lipase PS amano SD from *B. cepacia*.

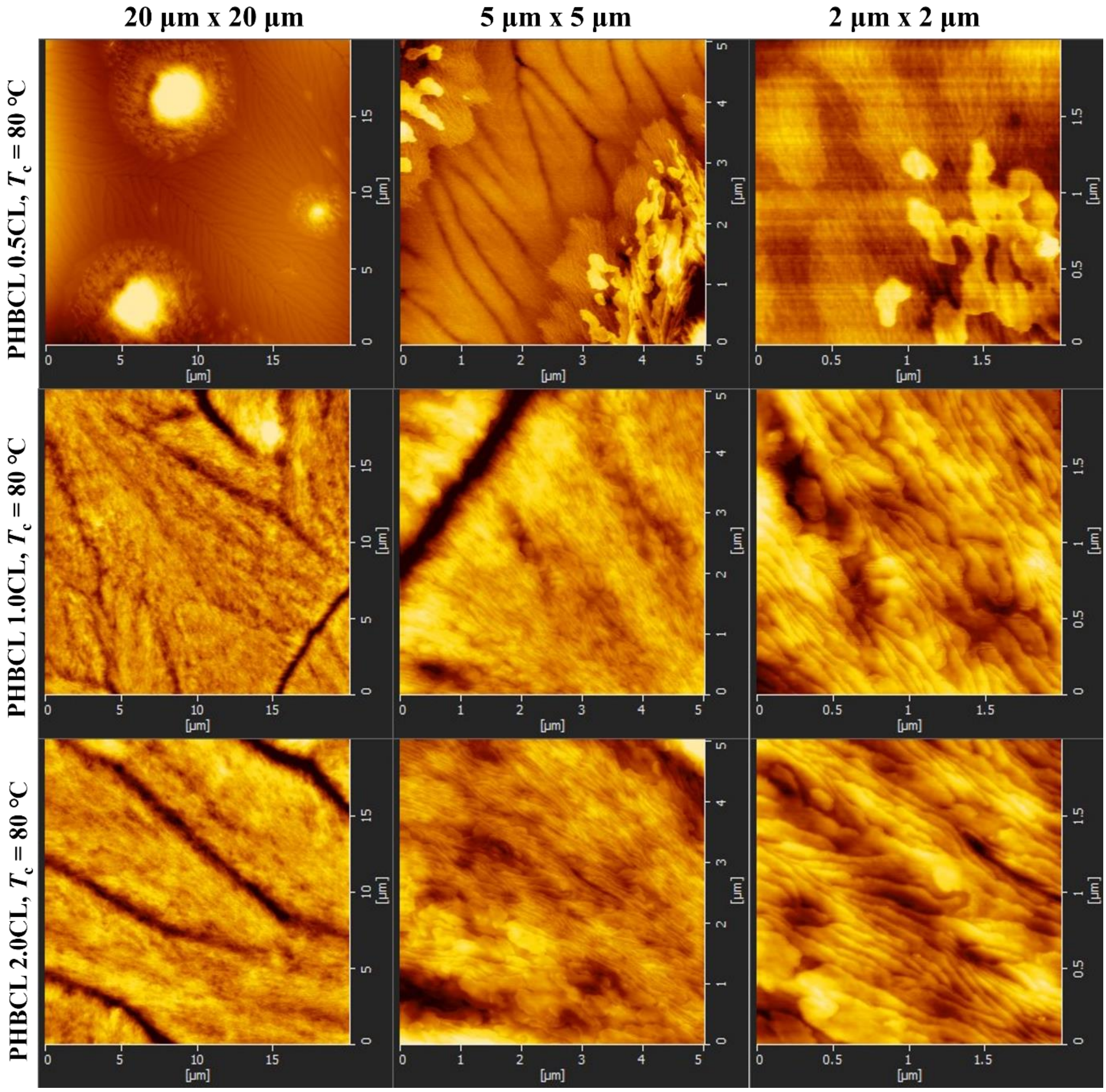

Figure S10. AFM height images for PHBCL diblock copolymers with varying PCL block lengths crystallized at 80 °C.

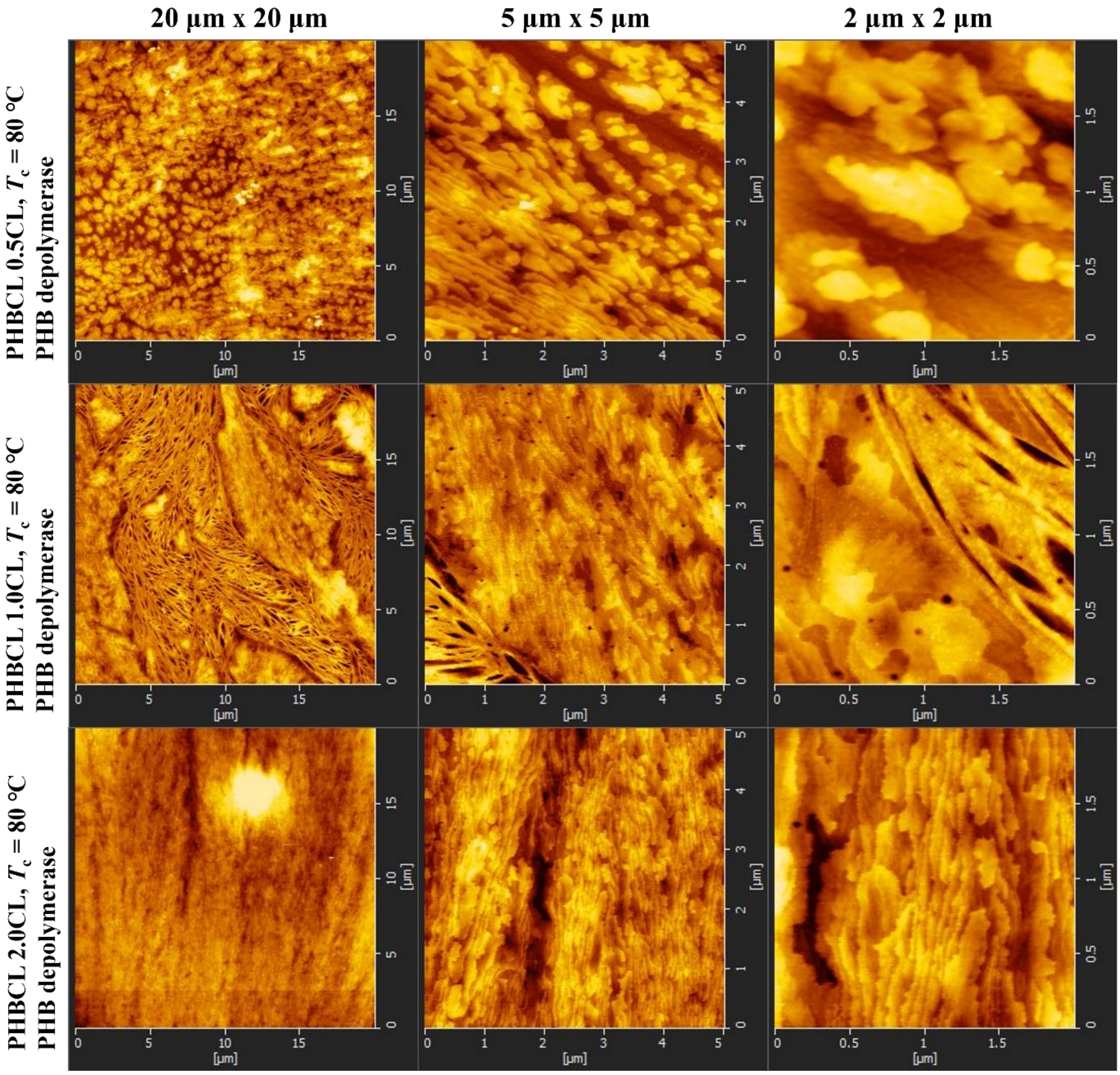

Figure S11. AFM height images for PHBCL diblock copolymers with varying PCL block lengths crystallized at 80 °C after 40 minutes exposure to PHB depolymerase from *R. picketti* T1.

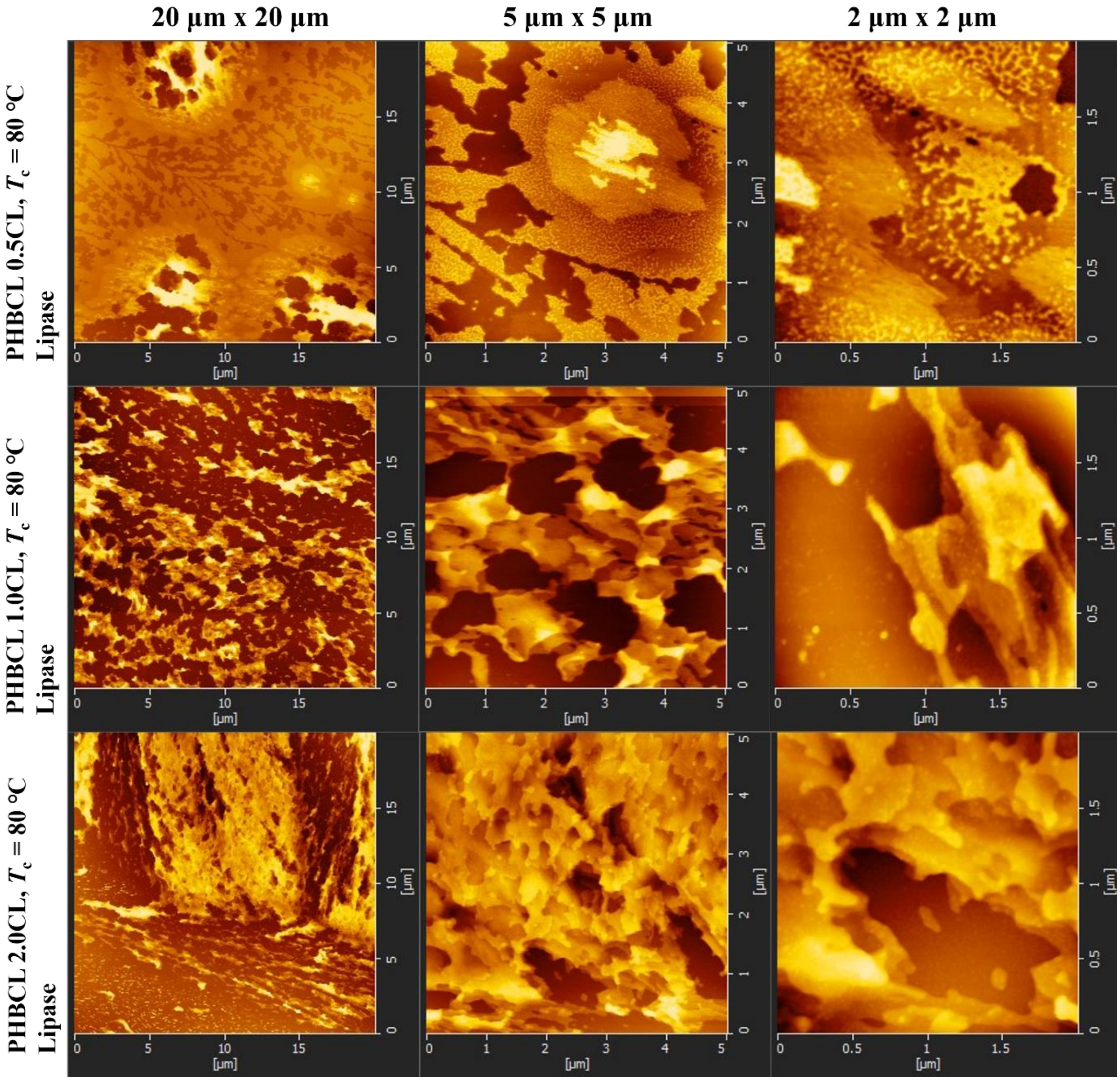

Figure S12. AFM height images for PHBCL diblock copolymers with varying PCL block lengths crystallized at 80 °C after 30 minutes exposure to lipase PS amano SD from *B. cepacia*.

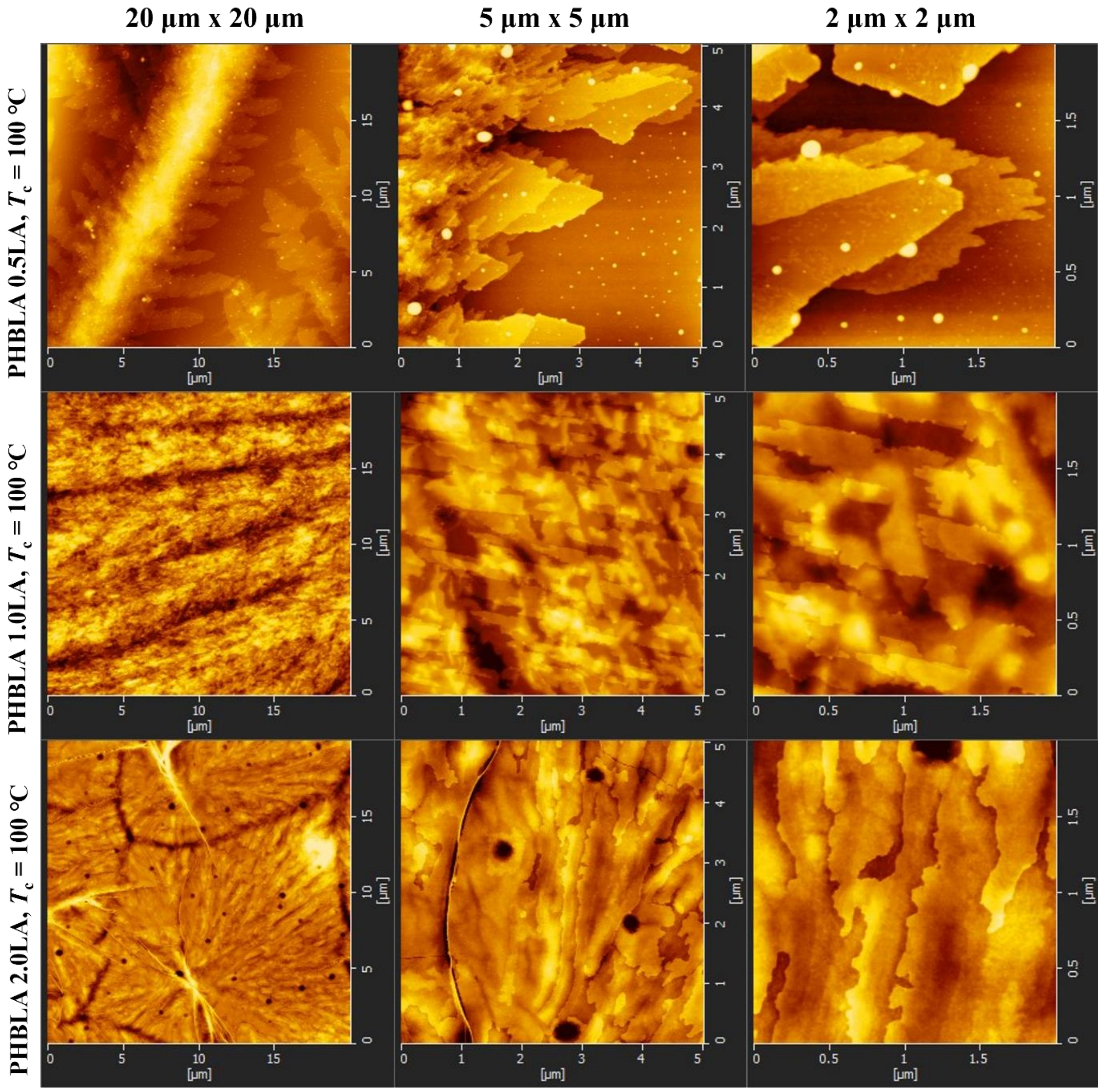

Figure S13. AFM height images for PHBLA diblock copolymers with varying PLA block lengths crystallized at 100 °C.

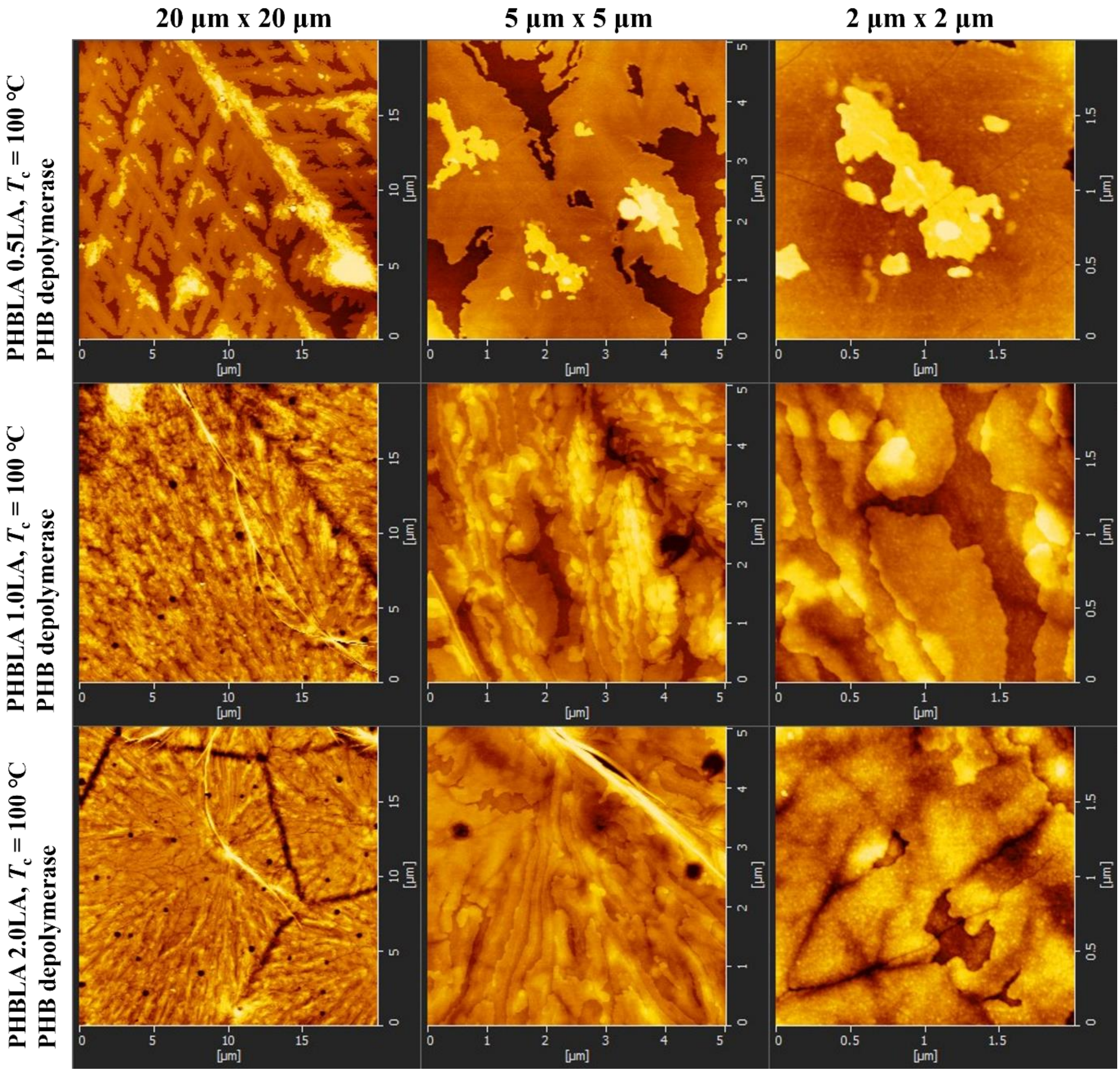

Figure S14. AFM height images for PHBLA diblock copolymers with varying PLA block lengths crystallized at 100 °C after 40 minutes exposure to PHB depolymerase from *R. picketti* T1.

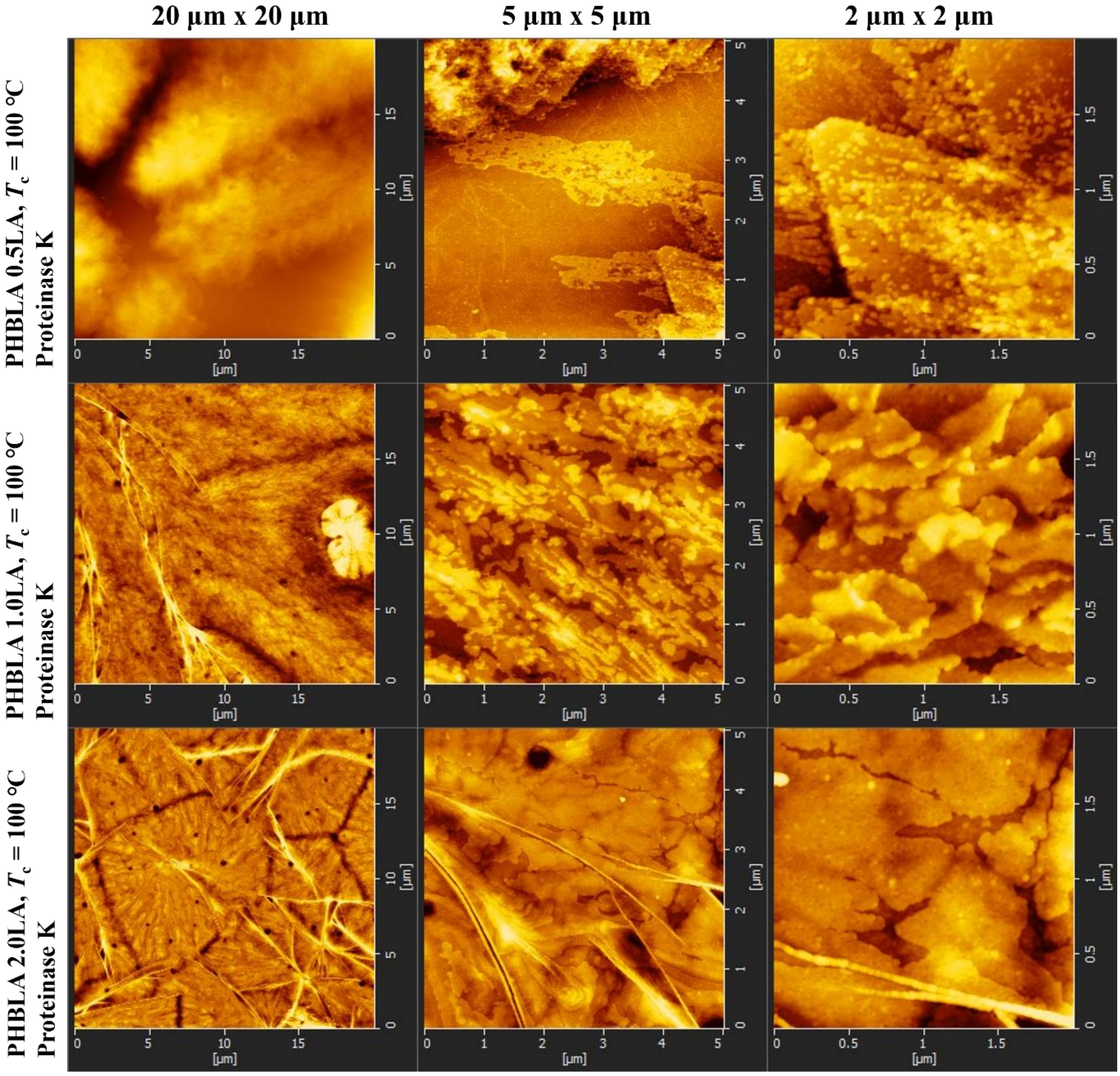

Figure S15. AFM height images for PHBLA diblock copolymers with varying PLA block lengths crystallized at 100 °C after 120 minutes exposure to proteinase K from *T. album*.

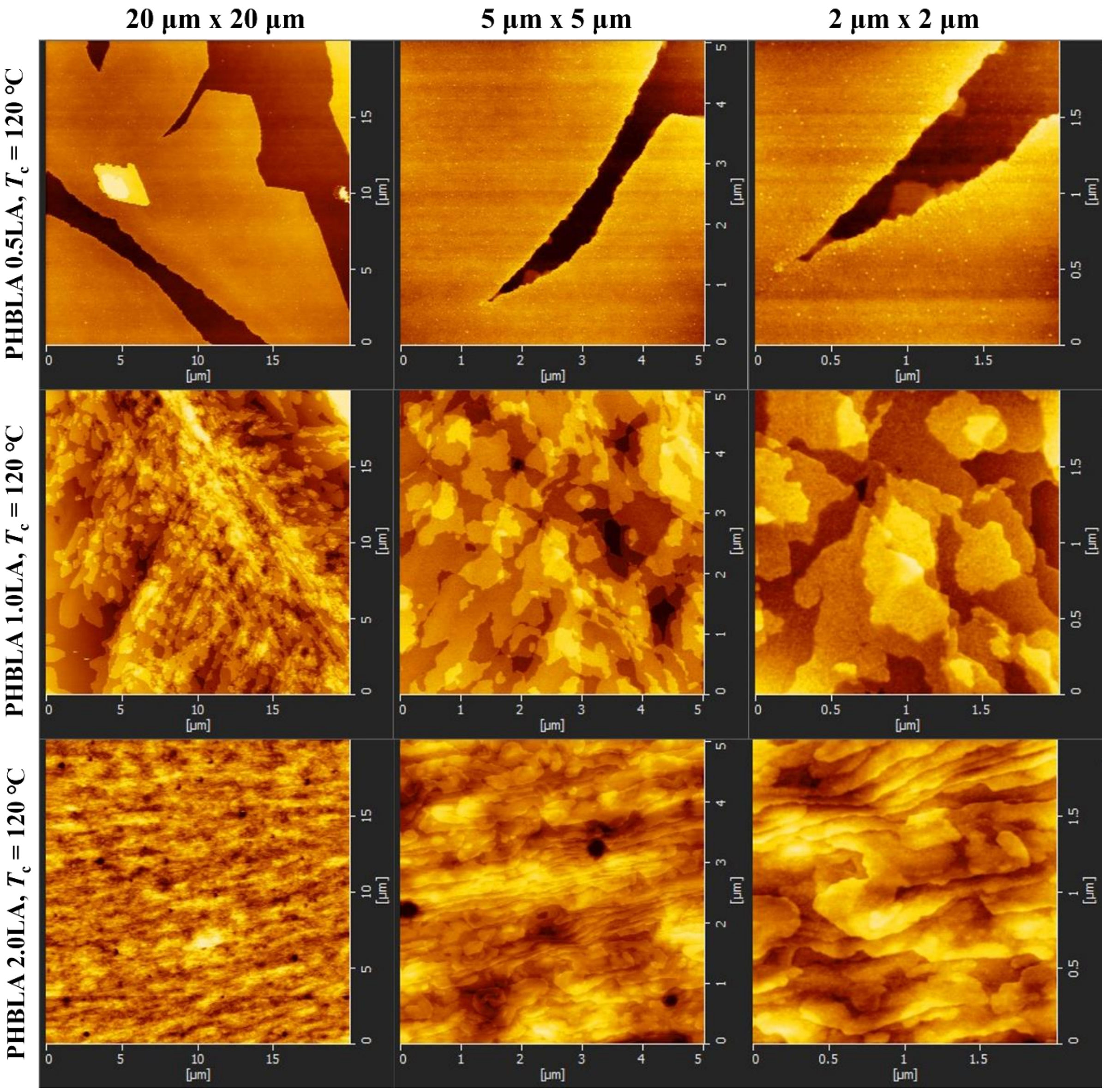

Figure S16. AFM height images for PHBLA diblock copolymers with varying PLA block lengths crystallized at 120 °C.

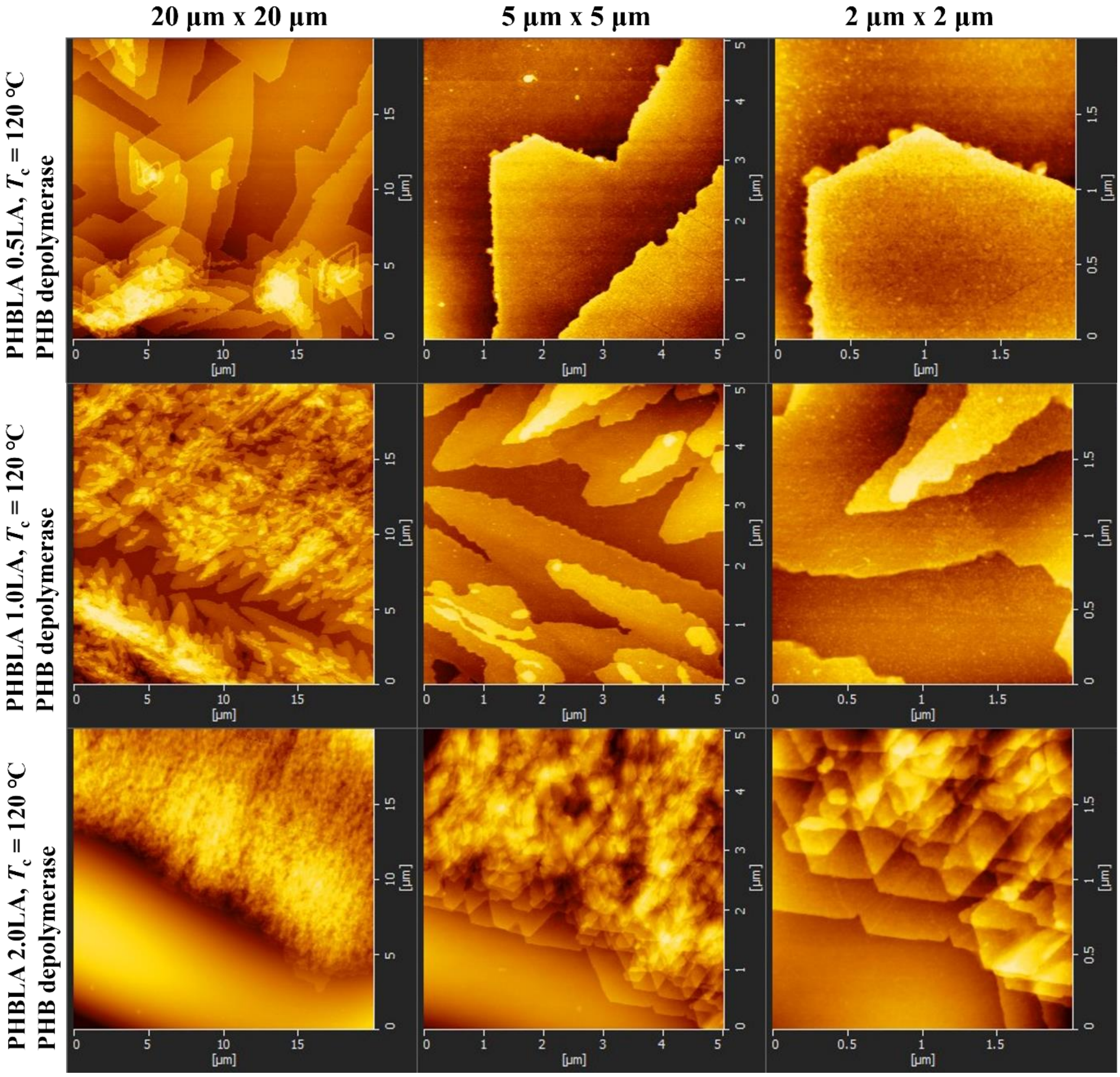

Figure S17. AFM height images for PHBLA diblock copolymers with varying PLA block lengths crystallized at 120 °C after 40 minutes exposure to PHB depolymerase from *R. picketti* T1.

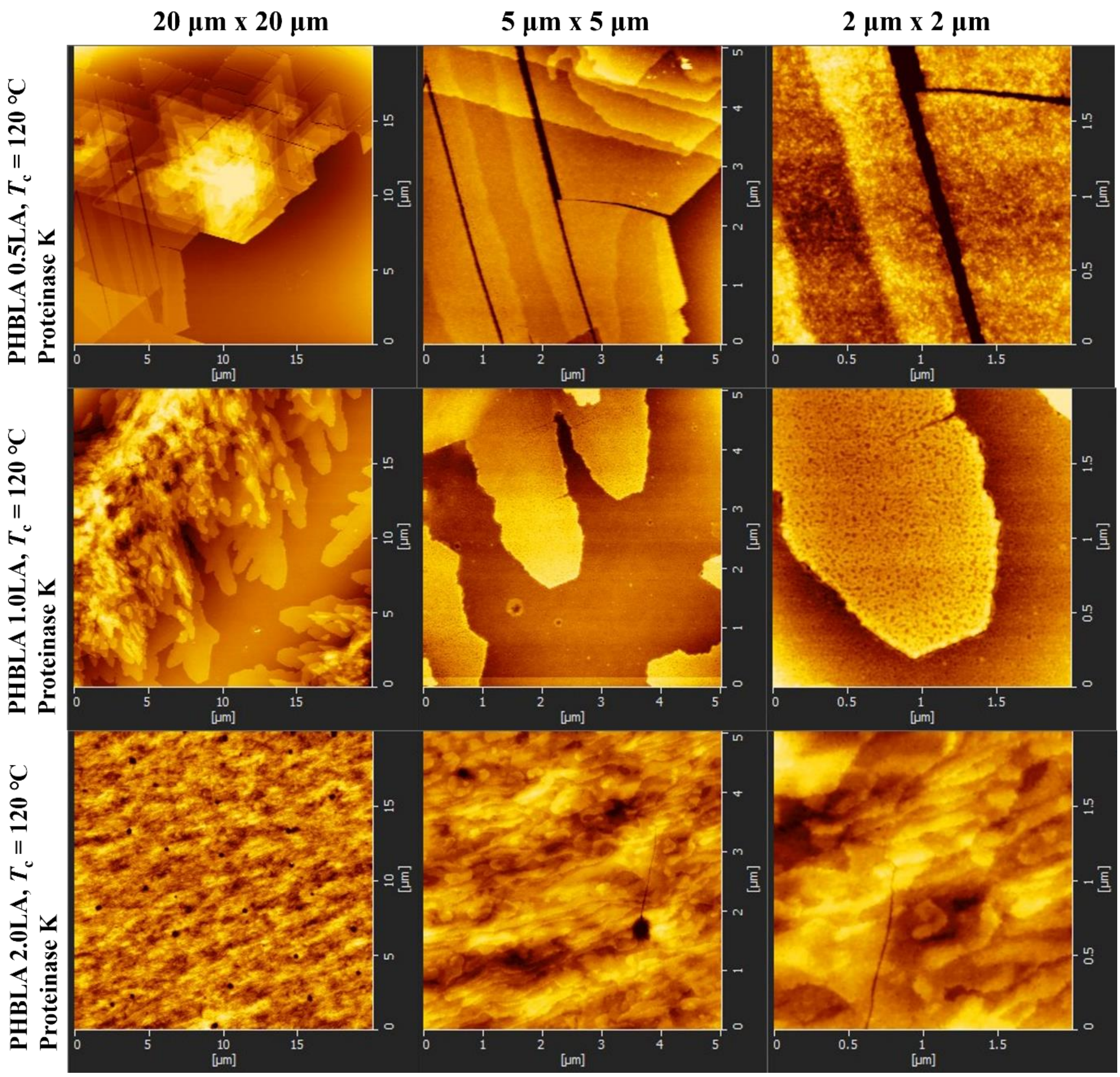

Figure S18. AFM height images for PHBLA diblock copolymers with varying PLA block lengths crystallized at 100 °C after 120 minutes exposure to proteinase K from *T. album*.

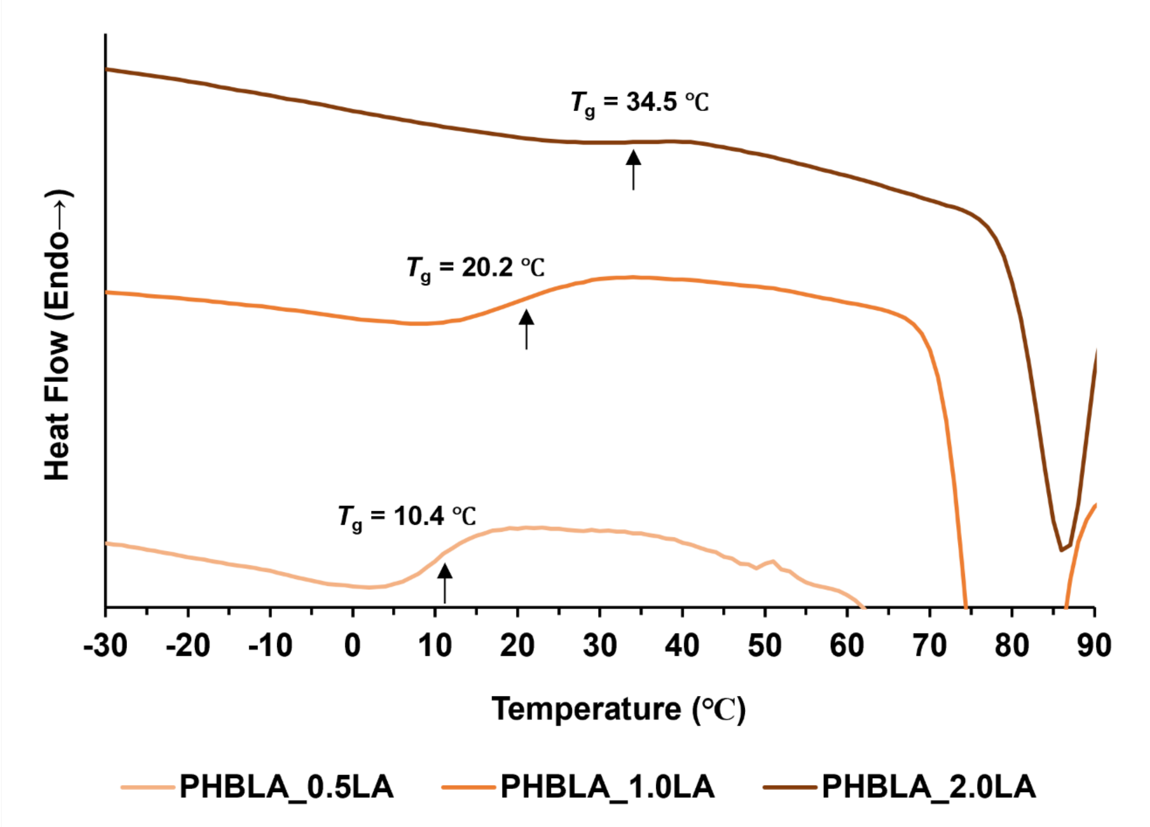

Figure S19. DSC 2nd heating thermograms enlarged to highlight the shifting T_g exhibited by PHBLA diblock copolymers.

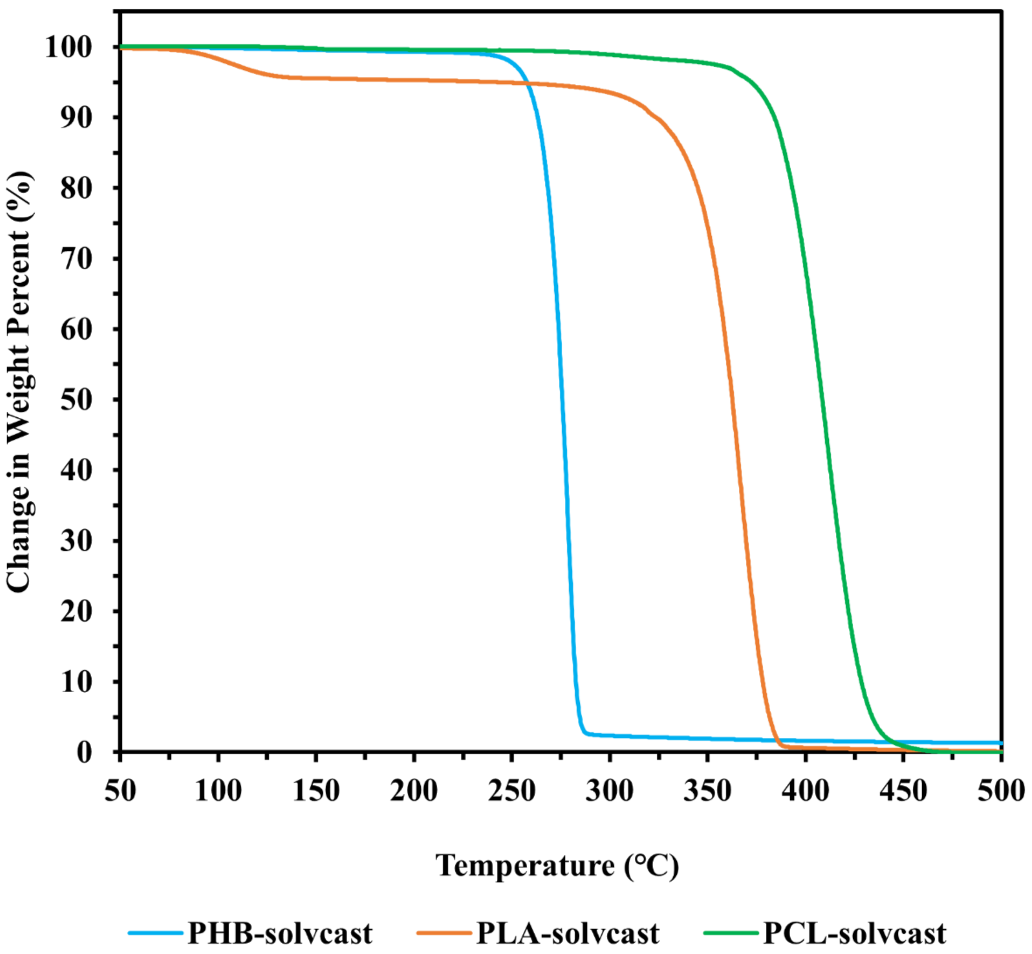

Figure S20. TGA curves for PHB, PCL and PLA homopolymers between 40 - 500°C using a 10°C/min heating regime.

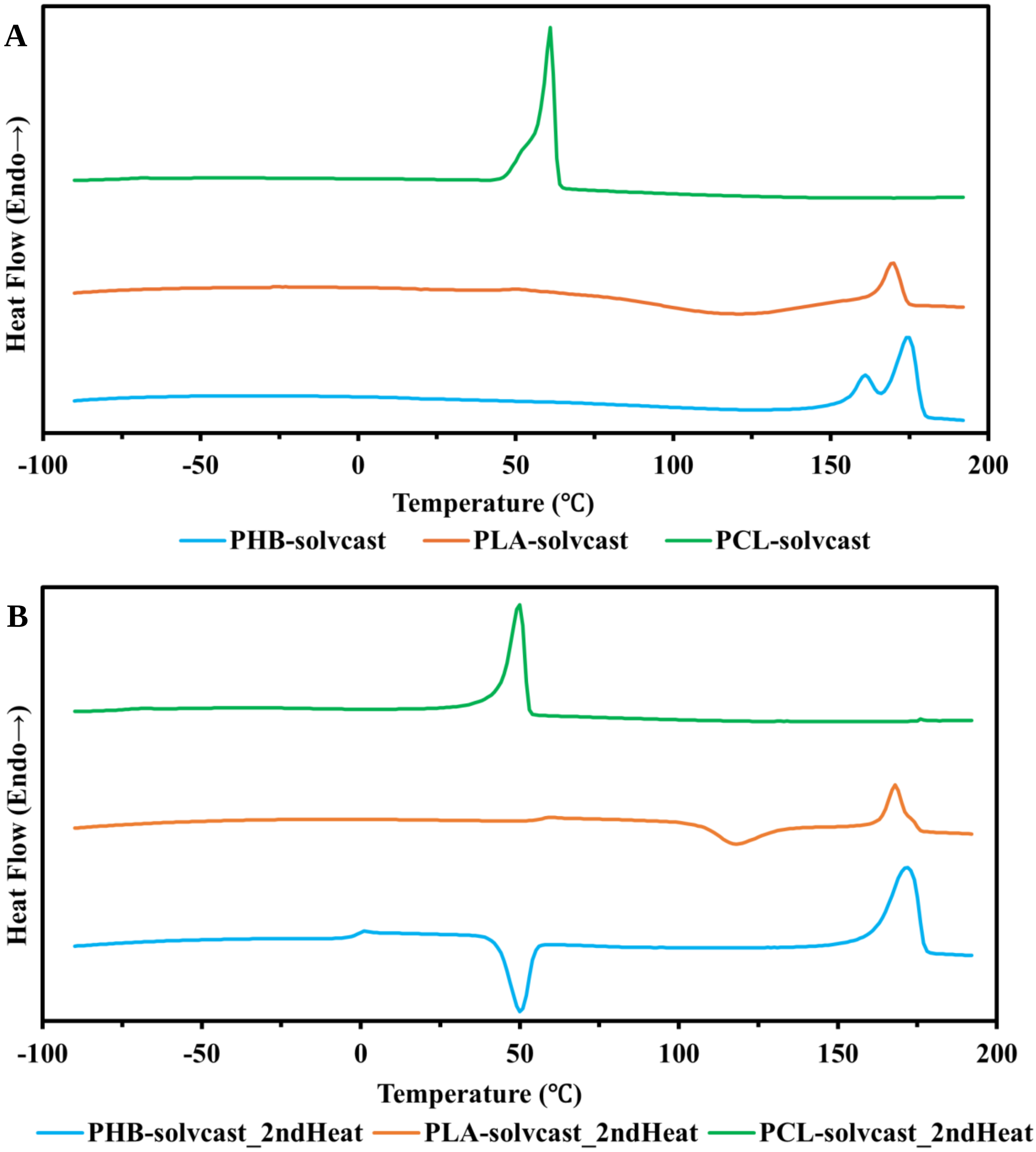

Figure S21. (A) 1st heating and **(B)** 2nd heating DSC thermograms showing relative change in heat flux with temperature for PHB, PCL and PLA homopolymers.

Table S3. Summary of thermal analysis of PHB, PCL and PLA homopolymers.

Homopolymer Sample	M_n (kg mol^{-1}) ^{a}	M_w (kg mol^{-1}) ^{a}	M_w/M_n ^{a}	T_m (°C) ^{b}	ΔH_m (J g^{-1}) ^{b}	T_g (°C) ^{c}	$T_{d5\%}$ (°C) ^{d}	$T_{d50\%}$ (°C) ^{d}	T_{dmax} (°C) ^{d}
PHB	174.2	430.3	2.47	160.9<br>174.7	10.7<br>44.0	-1.2	257.6	275.7	279.6
PCL	152.8	222.0	1.45	61.0	80.1	-72.4	372.1	408.5	410.9
PLA	144.1	219.8	1.53	169.6	-36.9	56.6	243.0	362.5	368.0

[a] Molecular weight data obtained from GPC, using polystyrene standards and $CDCl_3$ as the eluent.
[b] Melting temperature (T_m) and enthalpy of fusion (ΔH_m) were measured by DSC and taken from the first heating scan.
[c] Glass transition temperature (T_g) was measured by DSC and taken from the second heating scan.
[d] Temperature required for 5% ($T_{d5\%}$), 50% ($T_{d50\%}$) and complete (T_{dmax}) thermal decomposition data was measured by TGA.
